# *Plasmopara viticola* the Causal Agent of Downy Mildew of Grapevine: From Its Taxonomy to Disease Management

**DOI:** 10.3389/fmicb.2022.889472

**Published:** 2022-05-11

**Authors:** Kseniia Koledenkova, Qassim Esmaeel, Cédric Jacquard, Jerzy Nowak, Christophe Clément, Essaid Ait Barka

**Affiliations:** ^1^Université de Reims Champagne Ardenne, RIBP EA4707 USC INRAE 1488, SFR Condorcet FR CNRS 3417, Reims, France; ^2^School of Plant and Environmental Sciences, Virginia Polytechnic Institute and State University, Saunders Hall, Blacksburg, VA, United States

**Keywords:** *Plasmopara viticola*, grapevine, downy mildew, disease management, *Vitis vinifera*

## Abstract

*Plasmopara viticola* (*P. viticola*, Berk. & M. A. Curtis; Berl. & De Toni) causing grapevine downy mildew is one of the most damaging pathogens to viticulture worldwide. Since its recognition in the middle of nineteenth century, this disease has spread from America to Europe and then to all grapevine-growing countries, leading to significant economic losses due to the lack of efficient disease control. In 1885 copper was found to suppress many pathogens, and is still the most effective way to control downy mildews. During the twentieth century, contact and penetrating single-site fungicides have been developed for use against plant pathogens including downy mildews, but wide application has led to the appearance of pathogenic strains resistant to these treatments. Additionally, due to the negative environmental impact of chemical pesticides, the European Union restricted their use, triggering a rush to develop alternative tools such as resistant cultivars breeding, creation of new active ingredients, search for natural products and biocontrol agents that can be applied alone or in combination to kill the pathogen or mitigate its effect. This review summarizes data about the history, distribution, epidemiology, taxonomy, morphology, reproduction and infection mechanisms, symptoms, host-pathogen interactions, host resistance and control of the *P. viticola*, with a focus on sustainable methods, especially the use of biocontrol agents.

## Downy Mildew in Agricultural Systems

Downy mildews are primarily foliage blights caused by obligate biotrophic plant parasites which result in considerable economic losses on numerous crops. Plant diseases caused by oomycetes are separated into two types: those affecting plant parts in, or in contact with, the soil and those affecting aerial plant parts. Downy mildews infect young, tender green leaf, twig, and fruit tissues, causing severe losses in short periods of time. These infections can destroy 40–90% of plants in the field in optimal humidity and temperature (Toffolatti et al., 2018).

**Figure 1:**
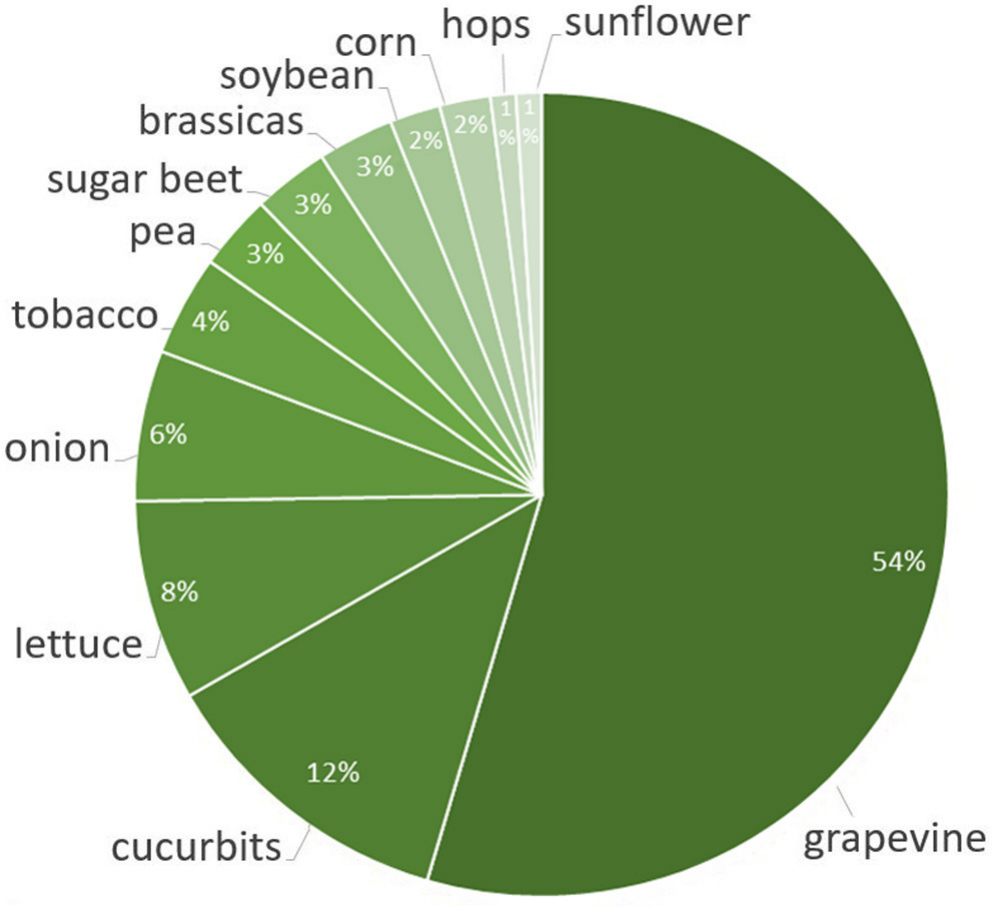
The most popular hosts of downy mildews. The color density corresponds to the rate of the crop damaged upon the total host range.

Downy mildews have caused spectacular and catastrophic epidemics on crops in the past, and many of them are still difficult to control despite the discovery of systemic fungicides which improved the ability to control fungal diseases. The grapevine downy mildew decimated the European viticulture and wine industry in the late 1800s. It had been endemic within North America, where native grapevine species developed fungal resistance. In 1878 it was introduced to southwest France through imported American grapevine rootstocks resistant to the phylloxera (Gessler et al., 2011). Within 5 years of its detection by Jules Emile Planchon, *P. viticola* had spread to all French, Italian, German and Swiss vineyards (Planchon, 1887). Grapevine downy mildew remained out of control until Pierre-Marie Alexis Millardet discovered the antifungal properties of copper sulfate in 1882 and invented the Bordeaux mixture in 1885 (Millardet, 1885; Gessler et al., 2011). Downy mildews have significant economic impact, as represented in Figure 1, namely grapes (54% of the total host range), cucurbits (12%), lettuce (8%), leek and onion (6%), tobacco (4%), peas (3%), brassicas (3%), sugar beet (3%), soybeans (2%), corn (2%), hops (1%), and sunflower (1%) (Gisi and Sierotzki, 2008).

Together with other widespread eukaryotes, namely diatoms and seaweeds, the fungal-like oomycetes belong to the kingdom Chromista (Stramenopila). They have mycelium containing glucans and cellulose, however, they have no cross walls excluding distinct living (cytoplasmic) hyphal portions from older portions from which the cytoplasm has been withdrawn. The phylum Oomycota includes several significant plant pathogens of economically important crop plants including necrotrophs in Pythium (Pythium ultimum), obligate biotrophs responsible for downy mildew (*Hyaloperonospora arabidopsidis, P. viticola*) and hemibiotrophs in Phytophthora (*P. sojae, P. capsica, P. infestans*) (Kamoun et al., 2015). Oomycetes belong to two orders, explicitly Saprolegniales and Peronosporales. The order Peronosporales includes mainly plant parasitic species a few of which are among the most economically impactful plants pathogens. More than 700 species within this group are downy mildew pathogens on a wide range of mono- and dicotyledonous plants (Thines and Choi, 2016). The most common oomycetes and downy mildews that they cause are listed in Table 1.

**Table 1:** The most common downy mildew genera, their hosts and control strategies.

**Genus**	**Most represented species**	**Hosts**	**Current control measures**	**References**
*Bremia*	*B. lactucae*	Lettuce	•Genetic resistance •Cultural practices •Chemical protection	Lebeda, 2002
*Hyaloperonospora*	•*H. arabidopsidis* •*H. parasitica* •*H. brassicae*	•*Arabidopsis thaliana* •Broccoli, •Radish, •Cabbage, Mustard, •Cucurbits	•Resistant genotypes •Crop rotation •Fungicide treatments •Reduction of humidity in greenhouses	Holub, 2008; Coates and Beynon, 2010
*Peronospora*	•*P. tabacina* •*P. belbahrii* •*P. destructor* •*P. effusa* •*P. manchurica* •*P. trifoliorum* •*P. sparsa* •*P. viciae*	•Tobacco •Basil •Onion, shallot •Spinach •Soybeans •Alfalfa, clover •Roses •Pea	•Crop rotation •Application of fungicides, biopesticides, SAR inducers •Cultivation of resistant varieties	Lim, 1989; Petatán-Sagahón et al., 2011; Wyenandt et al., 2015; Lyon et al., 2016; Thines and Choi, 2016; Kandel et al., 2019; Javed et al., 2021
*Peronosclerospora*	•*P. maydis and* •*P. philippinensis* •*P. sacchari* •*P. sorghi*	•Maize •Sugarcane •Sorghum	•Crop rotation •Disposal of crop debris •Cultivation of resistant varieties	Exconde, 1974; Bains and Jhooty, 1982; Jeger et al., 1998; Sullivan et al., 2010; Sireesha and Velazhahan, 2016
*Pseudoperonospora*	•*P. cubensis* •*P. humuli* •*P. cannabina* •*P. celtidis* •*P. urticae*	•Cucumber, Pumpkin, Zucchini, Squash, Melon, Watermelon •Hops •Cannabis •Chinese hackberry •Nettle (Urtica)	•Multiple application of both protectant and systemic fungicides •Cultivation of partially resistant varieties	Palti and Cohen, 1980; Choi et al., 2005; Savory et al., 2011; Cohen et al., 2015
*Sclerophthora*	•*S. macrospora* •*S. rayssiae*	•Rice, Sorghum, Wheat, Oat, Maize, Turf grass •Barley and Maize	•Cultural practices •Use of resistant varieties •Application of systemic fungicides •Biological control	Dick et al., 1984; Jeger et al., 1998; Putnam, 2007
*Plasmopara*	•*P. viticola* •*P. halstedii* •*P. euphrasiae* •*P. densa* •*P. crustosa* •*P. nivea* •*P. ribicola* •*P. obducens*	•Grapevine •Sunflower •Eyebright (Euphrasia) •Flax, Bartsia •Carrot, Chervil, Parsnip •Parsley •Currant •Impatiens	•Cultural practices •Seed treatments •Cultivation of partially resistant varieties •PGPR	Voglmayr et al., 2006; Thines et al., 2007; Voglmayr and Constantinescu, 2008; Gessler et al., 2011; Sharma et al., 2015

## Epidemics of Downy Mildews

The importance of downy mildews in agriculture has increased within the past 50 years, carried by the significant intercontinental trade and movement in grain, roots, and fruits. Downy mildew is one of the most damaging foliar diseases of grapevine, leading to organoleptic defects, productivity reduction and significant yield losses up to 75% in humid grapevine-producing areas worldwide (Darriet et al., 2002; Stummer et al., 2005; Jermini et al., 2010b; Gessler et al., 2011).

The importance of downy mildews in agriculture has increased within the past 50 years, carried by the significant intercontinental trade and movement in grain, roots, and fruits. After introduction from North America, this disease was a huge problem for European viticulture, causing epidemics in years with high humidity in the absence of sufficient control.

From 1907 to 1916, downy mildew reduced output of German vineyards by 33%, while significant periodic losses occurred in Italy in 1889, 1890, 1903, 1910, 1928, 1933 and 1934 (Müller, 1938). In 1915, 70% of the French grapevine production was destroyed by this pathogen. In 1930, 20 million liters of wine were lost in France (Cadoret, 1931).

In 1960, an epidemic of tobacco downy mildew spread to 11 countries and led to loss of 30% tobacco plants worldwide at a cost of $25 million. In the 1960s outbreaks occurred in Mexico, Israel, and the USA; in Texas alone, losses in 1969 reached $2.5 million with field incidence of disease up to 90%. In Taiwan several epidemics of sugarcane downy mildew have occurred from 1960 to 1964, leading to crop reduction of 70% (Payak, 1975).

In 1979, a devastating epidemic of this pathogen spread rapidly from Florida to Northeastern America and Canada causing losses to growers $200 million. In Cuba, the disease caused severe losses from 1978 to 1980 (Perez et al., 2003; Borrás-Hidalgo et al., 2010). Epidemics of Philippine downy mildew in 1974–1975 on maize and sugarcane cost the nation 8% of the total yield, leading to economic losses of $22.6 million (Exconde, 1974, 1976).

The first epidemics of cucurbits downy mildew occurred in 1984 on cucumbers and melon in Czechoslovakia. Damage was limited because of its appearance late in the growing season. In 1985 cucumber crops all over the Europe were heavily affected. The effect in Czechoslovakia was particularly devastating, with loss of 80–90% in cucumber yields. From 1986 to 1988, due to improved plant protection, economic losses caused by epidemics were lower, despite the high infection level. In 1989, however, losses in Czechoslovakia once again soared, reaching 80%.

African epidemics of sorghum and maize downy mildew during 1977, 1989, 1992, 1993, and 1995 led to crop losses ranging between 10 and 100% (Jeger et al., 1998). The sterility of systemically infected maize plants caused severe economic impact such as in Venezuela in the early 1970s (Frederiksen and Renfro, 1977). During the Indian epidemic in 1975, a harvest of 100,000 tons was discarded. Southern Nigeria was affected by maize downy mildew, where it caused losses of up to 15,000 tons and 12% of yield in 1991 and 1992.

In the decade since 2009, outbreaks of downy mildew in Central Europe have caused serious damage to hosts such as pumpkin, melon, watermelon, and Lagenaria, probably due to genetic changes in a virulence of *P. cubensis* (Lebeda, 1986; Lebeda and Schwinn, 1994; Cohen et al., 2015).

Grapevine downy mildew, caused by *P. viticola*, remains the most destructive downy mildew in Europe and the eastern half of the USA causing significant losses in grape production. The optimal temperature for *P. viticola* growth is 25°C; however rain is the main factor responsible for the epidemics. Downy mildew is one of the most damaging foliar diseases of grapevine, leading to organoleptic defects, productivity reduction and significant yield losses up to 75% in humid grapevine-producing areas worldwide (Darriet et al., 2002; Stummer et al., 2005; Jermini et al., 2010b; Gessler et al., 2011). After introduction from North America, this disease was a huge problem for European viticulture, causing epidemics in years with high humidity in the absence of sufficient control. In 1915, 70% of the French grapevine production was destroyed by this pathogen. In 1930, 20 million liters of wine were lost in France (Cadoret, 1931). From 1907 to 1916, downy mildew reduced output of German vineyards by 33%, while significant periodic losses occurred in Italy in 1889, 1890, 1903, 1910, 1928, 1933, and 1934 (Müller, 1938).

The growth of *P. viticola* is restricted in regions with low rainfall in the spring and summer and in northern regions, where sufficiently high temperatures are not reached in the spring. The downy mildew infects all green parts of the grapevine, including leaves, young stems and grapes, but leaves were found to be the main source of spores due to size of their surface, volume of cells for haustorial growth, stomatal amount and structure and the lack of protection against invasion of the pathogen. The disease is characterized by oily patches on the upper face of leaves that develop a dull green or yellowish color while the lower face exhibits a white growth. Infection of *P. viticola* causes leaf discoloration, necrosis and defoliation, which together reduce nutrient composition, sugar accumulation in berries, capacity for buds overwintering and harvest amount (Underdown et al., 2008). When infected, young berries become brown and are covered by white powder resulting from sporulation. As berries ripen, they become less susceptible to the infection, but rachis infections can spread into older berries (Gessler et al., 2011).

Rotting with subsequent mummification of inflorescences, shoots, clusters, and berries are also present if control measures are ineffective.

**Figure 2:**
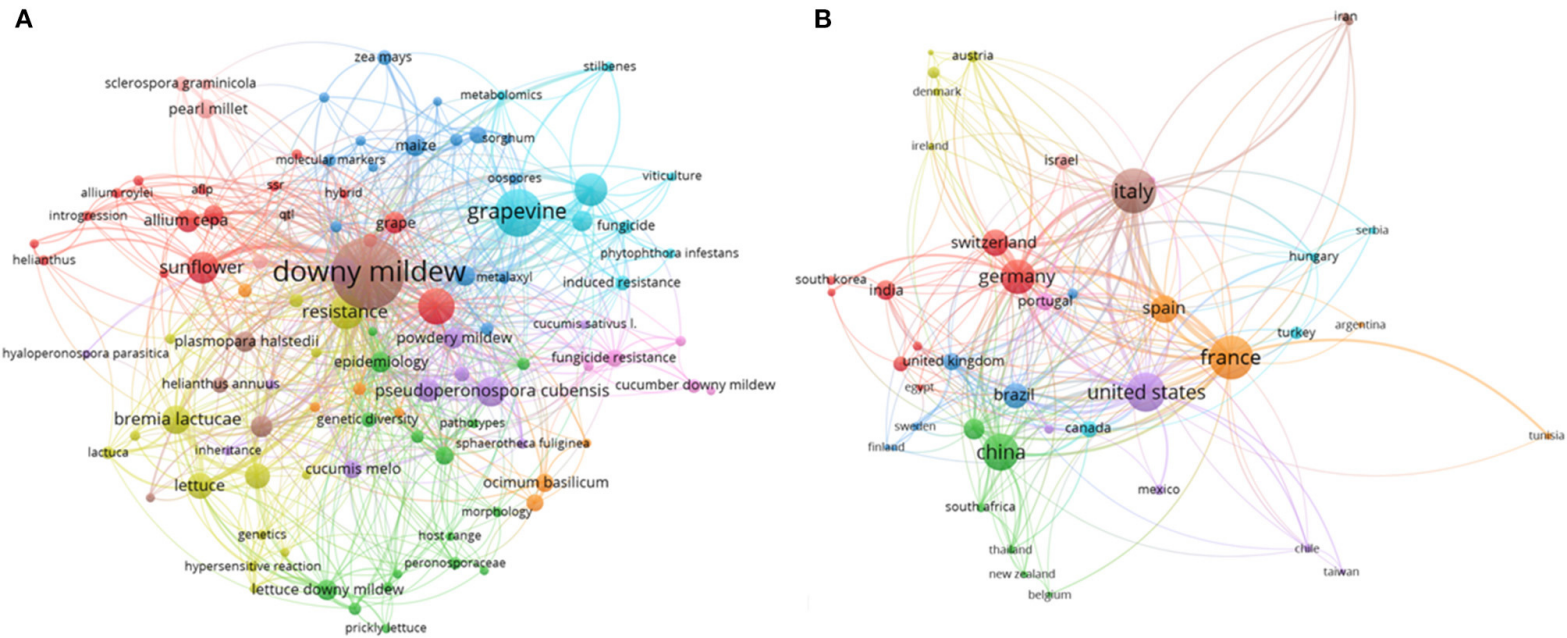
**(A)** Bibliometric analysis of the specific keywords used in the published literature related to downy mildew. Bibliographic data were extracted from the SCOPUS database using specific keywords “*Plasmopara viticola*” or “*P. viticola*” and “downy mildew” and “grapevine,” and the analysis include different bibliometric indices such as the most popular keywords, countries, and the top journals. For the network construction we used the VOSviewer processing software (https://www.vosviewer.com/ v1.6.9., Leiden University, Leiden, The Netherlands). **(B)** Publimetry related to downy mildew across the world. The network analysis, constructed using the VOSviewer software, includes the most popular countries.

In this review, bibliographic data were extracted from the SCOPUS database (https://www.scopus.com/) using specific keywords “*Plasmopara viticola*” or “*P. viticola*” and “downy mildew” and “grapevine,” from which 1649 documents were obtained. The bibliometric analysis was constructed using different bibliometric indices, including the most specific keywords used in the published literature related to downy mildew, countries, and the top journals. For the network construction, bibliometric analysis was constructed using the VOSviewer processing software (v1.6.9., Leiden University, Leiden, The Netherlands). The network analysis shows the worldwide distribution of related articles to downy mildew which help to highlight the relationships between countries, authors, and keywords found to get a comprehensive perspective of the current research of this area (Figure 2A). The analysis illustrated that most research conducted on downy mildew was demonstrated in the United States, China, France, Germany, Switzerland, and Italy (Figure 2B). It was noted that most articles addressed induced disease resistance, epidemiology, and control (Figure 2A).

## Taxonomic Evolution of Downy Mildew

**Figure 3:**
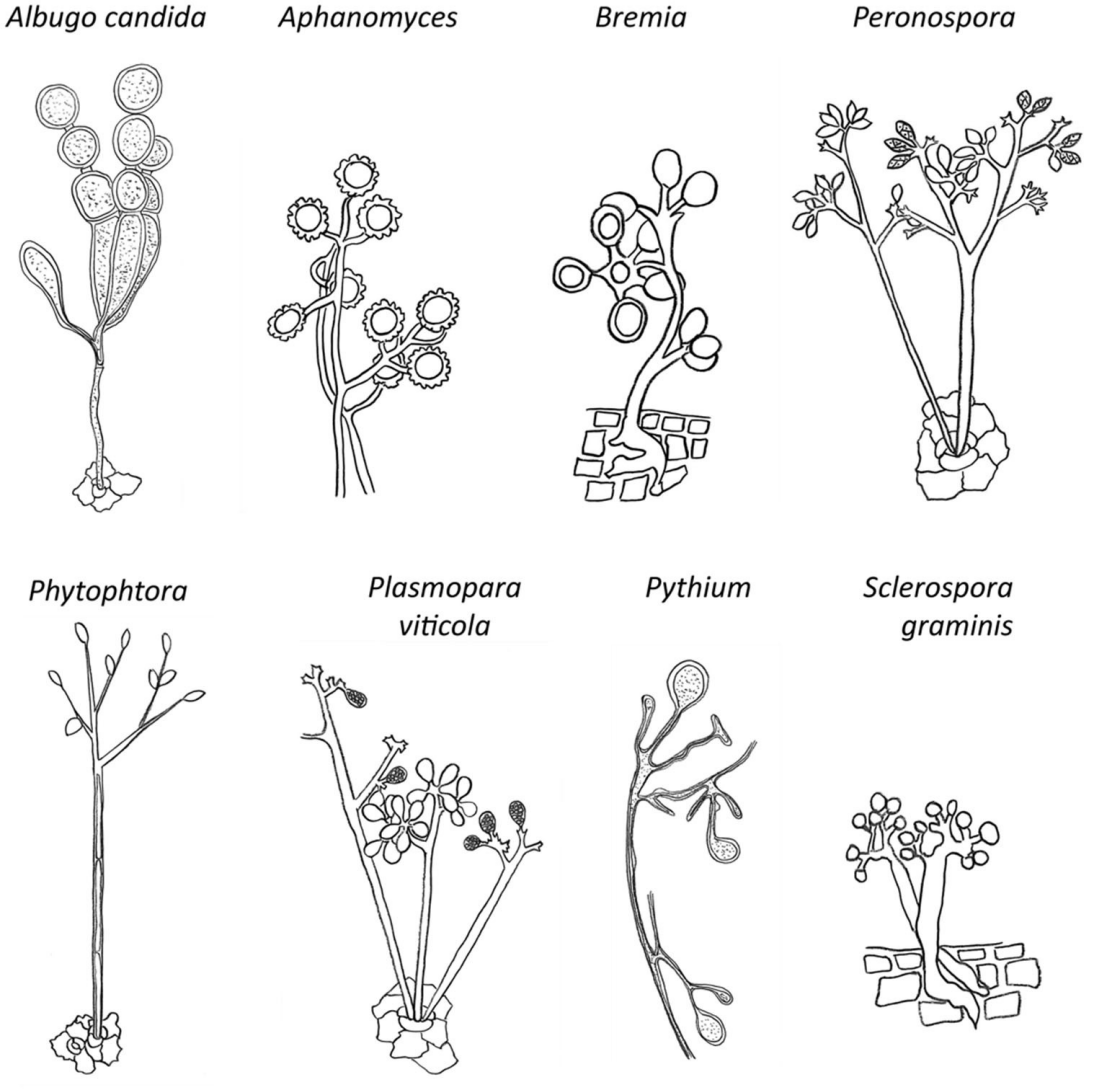
The most popular oomycetes that cause disease in plants. Morphological structures represented include length, branching and septation of conidio- or sporangiophores; conidial/sporangial size, shape, position, amount, and type of connection.

The downy mildews are similar to fungi in many ways, however, they do not constitute part of a monophyletic development of fungi within the eukaryote domain (Money, 1998). Downy mildews (*Peronosporaceae*) are a morphologically diverse group of oomycetes, mainly united by obligate parasitism in combination with presence of conidio- or sporangiophores with determinate growth. This group belongs to the orders Peronosporales and Sclerosporales in the class Peronosporomycetes, within the kingdom Straminipila, mainly based on morphological features of conidia/sporangia such as branching of conidio-/sporangiophores or shape of terminal branches (Figure 3) (Dick, 2002; Lebeda et al., 2008; Spencer-Phillips et al., 2016).

Varying approaches to classification have resulted in highly different numbers of accepted species depending on the criteria used, probably caused by the narrow host ranges because of an obligate parasitism, which represent genetically distinct species. In contrast, when morphology was used as a primary criterion for species definition, only few genetically heterogeneous species were defined (Hall, 1996). Morphological species concept, or Morphometric concept, is based on morphological characteristics such as size, shape, color of spores, oogonia and antheridia, the degree, kind of branching and length of sporangiophores (Figure 3). Using this phenetic approach, Oomycetes were divided into 3 subclasses: Saprolegniomycetidae, Rhipidiomycetidae, and Peronosporomycetidae, the latter including downy mildews (Dick et al., 1984; Dick, 1995).

With the availability of molecular phylogenies this subdivision of oomycetes was later confirmed (Dick et al., 1999; Riethmüller et al., 2002). Using this approach, Constantinescu showed that several *Asteraceae* pathogens, previously related to *Peronospora*, had in fact consistently different morphology and were placed in a new genus *Paraperonospora* (Constantinescu, 1989). In spite of an importance of morphological characteristics in classification, there is evidence that many characteristics of downy mildews can vary in the same species depending on the host, environmental conditions, nutrient media and reproductive features such as presence of heterothallism (Michelmore, 1981; Gustavsson, 1987; Johnson, 1989).

The Eco-physio-phenetic concept, introduced by Skalický, combined an ecophysiological (biological) criterion of host specialization with morphological properties. Several species of *Peronospora* on the host family *Rosaceae* were delimited based on their host subfamilies, morphology of conidiophores and conidial size: *Peronospora rubi* on Rubus was separated from *Peronospora sparsa* on Rosa, and *Peronospora potentillae* was separated from *Peronospora potentillae-reptantis*. Like in a biological species concept, Skalický did not control cross-infection experiments, so presence of host differences that had been used for species delimitation, was not proved (Skalický, 1983). Based on this approach, downy mildews have been divided into two sections, the Graminicolous and Eudicot-infecting downy mildews, based on mycelial characters, haustorial shapes and conidiophore morphology (Dick et al., 1984).

In 1992 Barr and Désaulniers conducted an ultrastructural analysis of zoospores applicable to systematics. They found a difference of zoospore flagellar apparatuses between *Phytophthora infestans, Phytophthora mirabilis*, and other *Phytophthora* spp., suggesting the presence of 2 groups, phylogenetically distinct from other *Phytophthora* species in a *Pythiaceae family* (Barr and Désaulniers, 1992). Ultrastructure of zoospores was firstly examined by transmission electron microscopy (TEM) in *Sclerospora graminicola*, where discharge papilla (a significant feature for identification, for example, a Peronosporales order) was found to take a part in zoosporogenesis (Lange et al., 1984). Surface ornamentation of conidia was described using scanning electron microscopy (SEM) on *Peronospora sordida* and *Peronospora statices*, where it was a distinguishing trait in addition to conidial lengths and breadths (Hall and Humphreys-Jones, 1989). A study using the SEM of three *Basidiophora* species demonstrated the occurrence of species-specific sporangial ornamentation, sporangiophore detachment scars and sporangiophore pedicels in *B. entospora* and *B. kellermannii* (Barreto and Dick, 1991). In 2004 Spring and Thines discovered additional ultrastructural features by SEM: using a model of downy mildews, they found classical features like conidio-/sporangiophore branching or sporangial germination are not suitable, but structure of the ultimate branchlets and haustorial shape were approved to be phylogenetically informative (Spring and Thines, 2004). Different haustorial types in downy mildews were described as far back as in 1956, but they were neglected for classification until 2000, when they started to be used as a diagnostic for several lineages as *Hyaloperonospora, Pseudoperonospora, Plasmopara*, and *Bremia* (Fraymouth, 1956; Constantinescu and Fatehi, 2002; Riethmüller et al., 2002).

A few biochemical characteristics such as fatty acids composition also were used for classification of downy mildews. Spring and Haas justified distinction of the oomycetes from true fungi and suggested that fatty acid composition together with the host specificity could be a promising diagnostic trait for species classification in obligate biotrophs in absence of clear morphological differences (Spring and Haas, 2002).

With the advent of molecular systematics, use of only morphological features for classification was deemed to be too simplistic. Molecular phylogenetic investigations have enabled the evaluation of the species problem using new perspectives and have led to the shift from a morphological to a phylogenetic species concept. In the absence of stable and correct morphological characteristics, this concept is increasingly based on molecular evidence of reproductive isolation, a general tendency within the mycology in recent decades. It became possible to recognize morphologically similar cryptic species as distinct ones if reproductive isolation and genetic distinctness can be demonstrated. However, molecular evaluation of boundaries between species requires use of several molecular markers and thorough sampling in all the distribution area which is difficult especially for biotrophic oomycetes (Lebeda et al., 2008). In published works, the most popular technique was widely used ribosomal DNA (rDNA) internal transcribed spacer region (ITS) sequencing, probably because of its simplicity and high variability compared with other genic regions of rDNA like small or large-subunit rRNA. ITS length was found to be a potential marker for species differentiation: its size originates from repetitive elements, number, and length of which seems to be taxon-specific (Voglmayr, 2008).

Riethmüller et al. analyzed nuclear large subunit ribosomal DNA sequences of the Peronosporomycetes to investigate their phylogenetic relationships (Riethmüller et al., 2002). During their study, basal division of Peronosporomycetes into Peronosporomycetidae and Saprolegniomycetidae, as proposed before, was confirmed. Also, division between the Pythiales and Peronosporales on the one hand and the Saprolegniales, Leptomitales, and Rhipidiales on the other was well-supported as well as the placement of orders Saprolegniales and Leptomitales in the Saprolegniomycetidae. The Sclerosporales, Peronosporomycetidae, Pythiales and Peronosporales were found to be polyphyletic. The *Verrucalvaceae* were merged within the Saprolegniales; *Peronophythora* was merged with *Phytophthora*; *Bremiella* was transferred to *Plasmopara*; and *Phytophthora* was found to be closer related to Peronosporaceae than to Pythiales, which was confirmed in later studies (Dick et al., 1984; Riethmüller et al., 1999, 2002; Cooke et al., 2000; Hallin et al., 2018). Monophyly of the genera *Pseudoperonospora* and *Hyaloperonospora* were supported (Voglmayr, 2003). The classification of the graminicolous downy mildews such as *Sclerospora* and *Peronosclerospora* using molecular phylogenetic data did not support this group as a separate family and order or classification within the Saprolegniomycetidae, so Graminicolous downy mildews were placed within *Peronosporaceae* (Riethmüller et al., 2002; Göker et al., 2003, 2007). Genera *Hyaloperonospora* and *Perofascia* were segregated from the large genus *Peronospora;* owing to their polyphyly, three species of *Bremiella* genus were embedded within *Plasmopara* (Constantinescu and Fatehi, 2002; Göker et al., 2007; Thines and Choi, 2016). Based on phylogenetic analyses, genus *Rhysotheca*, introduced by Wilson in 1907 as closely related to *Peronospola viticola*, was merged with *P. viticola* (Wilson, 1907; Riethmüller et al., 2002).

**Figure 4:**
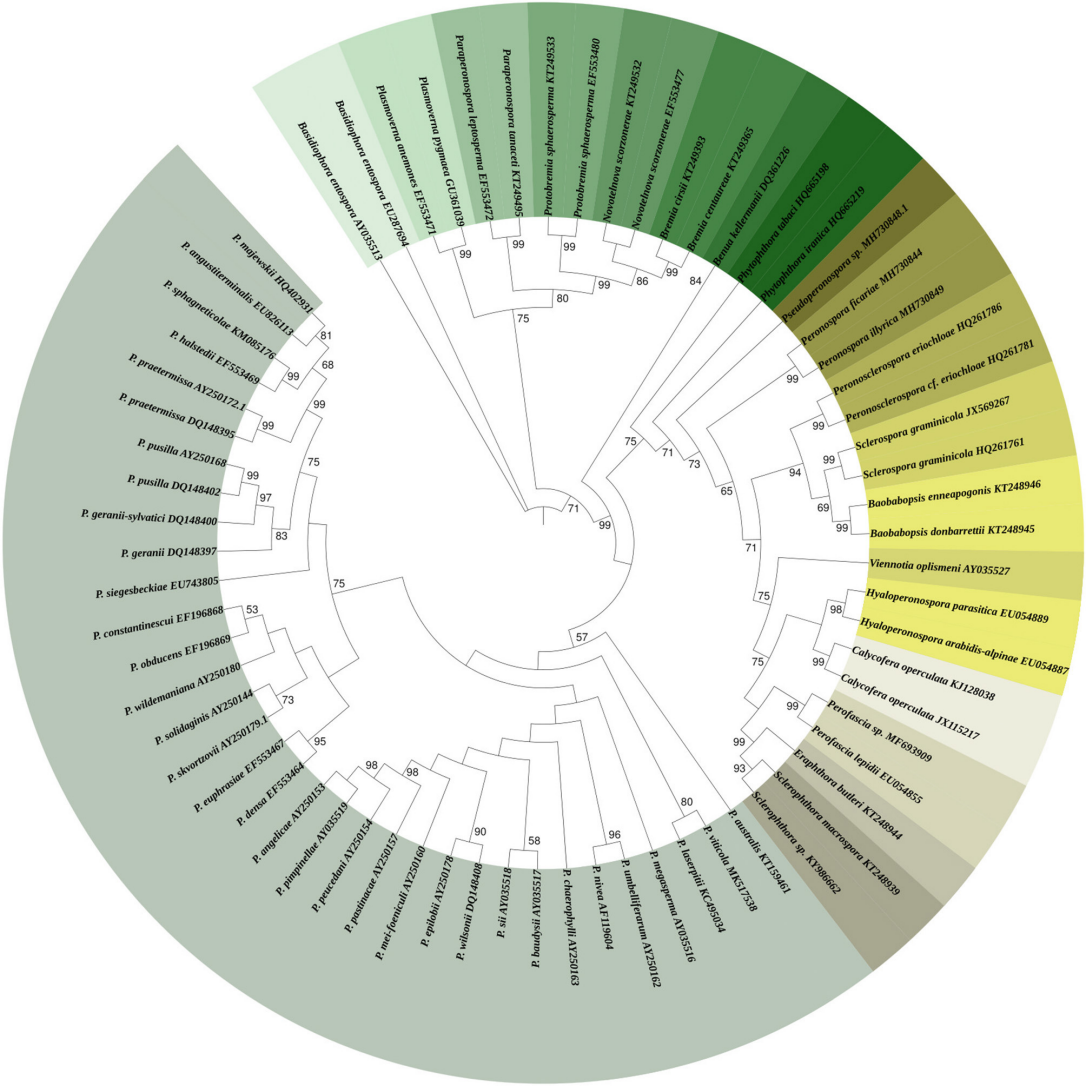
Phylogenetic tree represents different genera of *Peronosporaceae* family. For phylogenetic tree analysis, a large subunit of ribosomal RNA (LSU) gene sequences of the different genera of *Peronosporaceae* family were retrieved from the National Center for Biotechnology Information database (NCBI) (http://www.ncbi.nlm.nih.gov). Collected sequences were aligned to construct the phylogenetic tree. The tree was built using MEGA version X with neighbor-joining method. The dataset was boot-strapped 1,000 times. Values at nodes indicate bootstrap values out of 1,000 resampling.

To obtain insight into the phylogenetic relationships within the different genera of Peronosporaceae family, the large subunit of ribosomal RNA (LSU) gene sequences of the different genera of this family were retrieved from the National Center for Biotechnology Information database (NCBI) (http://www.ncbi.nlm.nih.gov) and aligned to construct the phylogenetic tree (Figure 4). The tree was built using MEGA version X with neighbor-joining method. The dataset was boot-strapped 1,000 times and values at nodes indicate bootstrap values out of 1,000 resampling.

**Figure 5:**
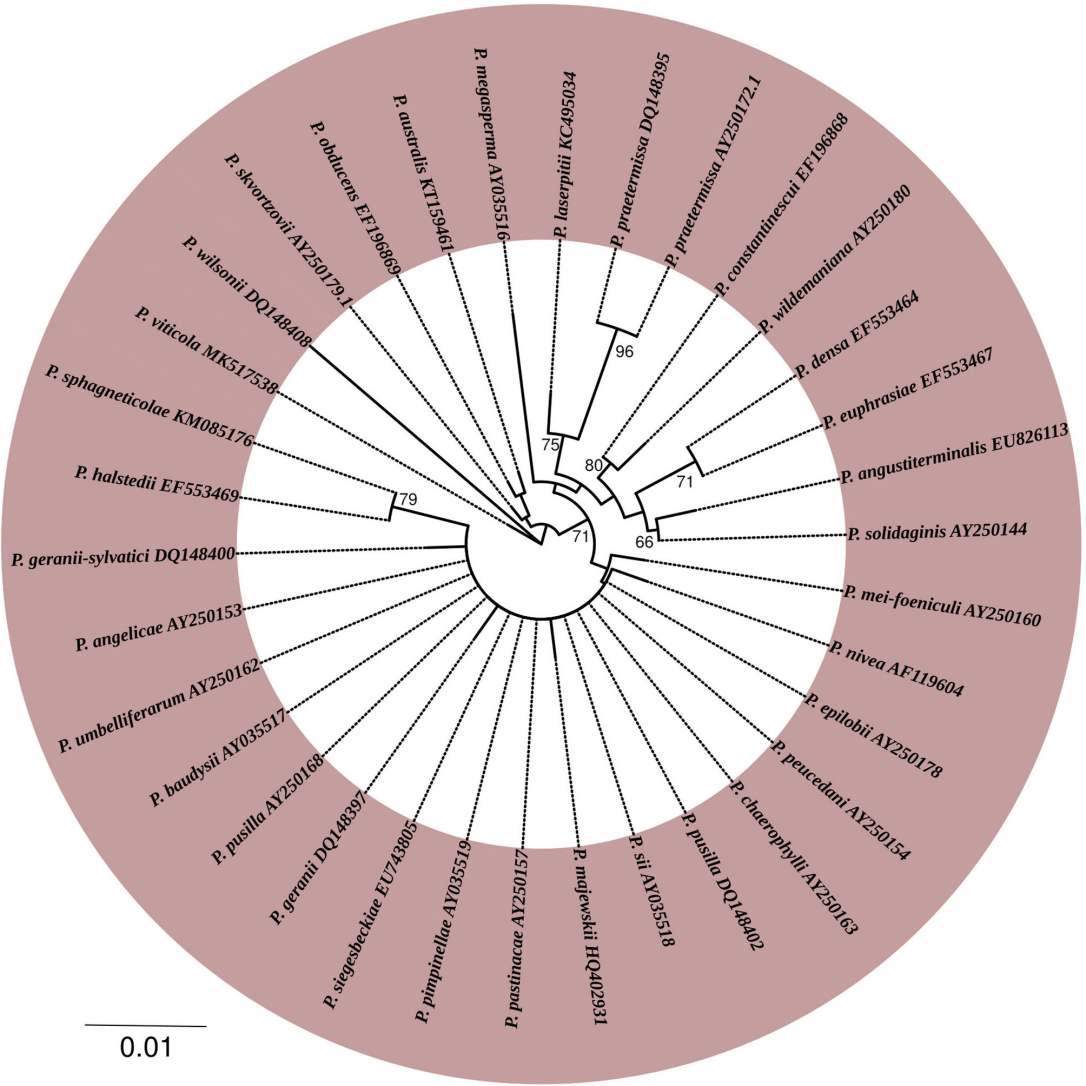
Phylogenetic tree represents different members of *Plasmopara* genus. The tree was built with MEGA version X using the large subunit of ribosomal RNA (LSU) gene sequences of the different members of *Plasmopara* genus. Values at nodes indicate bootstrap values out of 1,000 resampling.

*Plasmopara* was proved be a polyphyletic genus, so several *Plasmopara* and *Bremia* pathogens of *Poaceae, Cichorieae, Ranunculaceae*, and *Scorzonera* hosts were segregated into new genera *Viennotia, Poakatesthia, Graminivora, Protobremia, Plasmoverna*, and *Novotelnova. Plasmopara euphrasiae* was segregated from *Plasmopara densa* and *Plasmopara centaureae-mollis* was revised and synonymised with *Bremia centaureae* (Riethmüller et al., 2002; Göker et al., 2003; Voglmayr et al., 2004; Thines et al., 2006, 2007; Voglmayr and Constantinescu, 2008). Based on differences in partial sequence analysis of the nuclear ITS region, Spring et al. succeeded in differentiating between certain pathotypes of *Plasmopara halstedii* originating from different geographic regions (Spring and Zipper, 2006). Molecular studies of *Pseudoperonospora* proved the conspecificity of species from different hosts: *Pseudoperonospora humuli* was synonymized with *Pseudoperonospora cubensis* based on identity of ITS sequences and morphology of both pathogens; later multiple host shifts from Cannabinaceae to Cucurbitaceae confirmed the conspecificity (Gregory, 1915; Choi et al., 2005). In the current review, a phylogenetic tree (Figure 5) harboring the different validated members of *Plasmopara* genus was constructed using the large subunit of ribosomal RNA (LSU) gene sequences. The tree was built using he tree was built using MEGA version X and the dataset was boot-strapped 1,000 times. Values at nodes indicate bootstrap values out of 1,000 resampling.

Molecular phylogenetic analyses showed a close phylogenetic relationship of downy mildews to the genus *Phytophthora*, suggesting an origin as a stem from different *Phytophthora* groups (Riethmüller et al., 1999; Cooke et al., 2000; Voglmayr, 2003). Later in a multigene (five genes) study Göker et al. found downy mildews to be monophyletic (Göker et al., 2007). Analyzing this five gene dataset by different methods of phylogenetic reconstruction, Göker found indications downy mildews are not monophyletic, and one of *Phytophthora* group was embedded within the downy mildews clade (Göker and Stamatakis, 2006). The additional data from other gene regions and taxa seems to be necessary to achieve a correct phylogeny.

## Plasmopara Viticola

### Taxonomy

Genealogies in *P. viticola*, an oomycete endemic to North America on wild and cultivated grapevines, have suggested the existence of four cryptic species, with different levels of host plant specialization varying from mild to complete host dependence. *P. viticola* (Berk. & Curt.) Berl. & de Toni, belonging to the order of Peronosporales and the family of Peronosporaceae was firstly collected by American botanist and mycologist Lewis David de Schweinitz in 1834 and it was classified as *Botrytis cana* (Schweinitz, 1834). In 1848 Henry William Ravenel in co-authorship with Miles Joseph Berkeley, re-classified *B. cana* as *Botrytis viticola* (Hendrickx, 1948). The German microbiologist Anton De Bary described in 1863 its both reproduction cycles and placed it in a new genus *Peronospora* as *Peronospora viticola* (De Bary, 1863). Then, Schröder found in 1886 differences between several *Peronospora* members and introduced a new genus of *Plasmopara* (Schröter, 1886). In 1888 Berlese and de Toni, using Schröder's classification system, gave this pathogen its current name of *Plasmopara viticola* (Berlese and De Toni, 1888).

### Morphology

Grapevine downy mildew, like many oomycetes, possess a filamentous vegetative body called a mycelium that is formed by dichotomously branching hyphae, which vary in diameter from 1 to 60 micrometers depending on a development stage of the pathogen. They grow in intercellular space of a plant in a coenocytic manner, i.e., they contain several nuclei without the formation of cross-walls that divide each hypha into separate cells in septate fungi. Haustoria project from the hypha to uptake nutrients from the host cells. Cell walls of oomycetes consists of cellulose, glucans, small amounts of chitin and hydroxyproline, differing from the true fungi (Bartnicki-Garcia, 1968; Werner et al., 1968; Bakshi et al., 2001). Cell wall-degrading enzymes are important in pathogen degradation; they are synthesized by the host plant specifically to target and break down cell wall components.

Mycelium produces sporangiophores that arise through stoma and produce lemon-shaped sporangia at their tips. This branching is perpendicular and monopodial, which means that the growth of the main sporangiophore branch continues while the lateral remains fixed, until 4–6 branches with 2 or 3 secondary branches are formed. Ultimate branches called sterigmata are mostly trichotomous in *Plasmopara* genus and have distinct annuli at their tips. Sporangia are sacs that release moving thin walled ellipsoid zoospores that are 15–30 μm in diameter and have two heterokont flagella. The anterior flagellum is longer and possesses 2 symmetric rows of tubular, tripartite hairs called mastigonemes, while posterior one is shorter and smooth (whiplash). Oomycetes also produce large, 25–50 μm in diameter, thick-walled spherical sexual spores called oospores.

### *P. viticola* Genome

#### Genome Architecture

To further understand the *P. viticola*-host interactions at the genome level, Dussert and collaborators sequenced the genome of *P. viticola* strain INRA_Pvit_2, obtaining a genome size of 92.9 Mb assembled into 358 scaffolds, using PacBio long-read technology (Dussert et al., 2016). Other isolates including JL-7-2 and PvitFEM01 have been sequenced and recorded online in the NCBI (Table 2). The genomes of different strains were sequenced using the methods summarized in Table 2; their sizes span from 83 to 101 Mb. The genome characteristics as well as the project information of different isolates of *P. viticola* are presented in Table 2.

**Table 2:** Project information and genomic features of draft genome sequences of different isolates of *P. viticola*.

**Property**	**Strain**	**INRA_Pvit_2**	**JL-7-2**	**PvitFEM01**
Sequencing technology		PacBio	Illumina HiSeq; PacBio	Illumina
Mean coverage		185.0x	207.0x	164.0x
Assembly method		PBcR v. wgs8.3rc2	AllPaths v. DEC-2015; PBJelly2 v. MAY-2016	Ray v. JANUARY-2014
Assembly level		Scaffold		
BIOPROJECT		PRJNA329579	PRJNA361333	PRJNA380033
BioSample		SAMN05415085	SAMN06231250	SAMN06627059
Source material identifier (host)		Leaves (*V. vinifera*)	Leaves (*V. labrusca*)	Leaves and spores (*V. vinifera*)
Project relevance		Agriculture		
Geographic location		France: Blanquefort	China: Jilin province	Italy: San Michele all'Adige
Size		92.94 Mb	101.30 Mb	83.54 Mb
G+C content (%)		44.80	37.50	42.40
Number of scaffolds		358	2,165	57,890
Scaffold N50		706,521	172,266	4,645
Scaffold L50		38	172	3,777
Number of contigs		374	23,193	65,120
Contig N50		666,562	14,258	2,161
Contig L50		41	1,201	7,173
GenBank ID		MBPM00000000	MTPI00000000	NBAH00000000
References		Dussert et al., 2016, 2019	China Agriculture University	Brilli et al., 2018

#### *P. viticola* Effectors

Numerous plant pathogens may release effector proteins to overcome the plant's immune defense response. The function of effector proteins in the pathogenic process has been widely dissected in bacteria. Effectors may have a crucial function at the first encounter between the plant and the pathogen to develop a compatible interaction. Oomycetes use an array of effectors translocated in intra- and extracellular plant tissues to modulate the plant metabolism to their benefit and promote infection. The ability to liberate effector proteins, which may penetrate plant cells and control host processes, is a central determinant of a host–pathogen interaction outcome (Casagrande et al., 2011). As effectors are produced by the pathogen, they elicit a host-translocation signal recognized by the host cells. Two primary classes of host translocated effectors have been identified: cytoplasmic RXLR targeting subcellular plant compartments and apoplastic LFLAK, secreted into the plant extracellular space. During independent genome sequencing of such pathogens as *Phytophthora, Pythium, Hyaloperonospora arabidopsidis* and *P. viticola*, several 100 intra- and extracellular effectors have been found (Rehmany et al., 2005; Haas et al., 2009; Mestre et al., 2012; Perazzolli et al., 2012; Chen et al., 2021).

RXLR effectors are the most studied group of oomycotic effectors; they are small AVR proteins with conserved N-terminal amino acid motifs consisting of arginine, any amino acid-leucine and leucine. Evidence points to the potential for RXLR effectors to drive plant cell death following recognition by the corresponding R proteins or unknown mechanisms (Huang et al., 2019). Many of RXLR effectors have a second EER-motif (glutamic acid-glutamic acid-arginine optional amino acid motif) participating in the transport from the pathogen to its host, and at varying distance C-terminal to RXLR motif (Stassen and Van Den Ackerveken, 2011; Liu et al., 2021). Both RXLR and EER motifs are needed to access host cells (Kale and Tyler, 2011). However, even while the RXLR motif was reported as responsible for effectors entering the host cell, its biological role remained controversial (Ellis and Dodds, 2011; Wawra et al., 2013, 2017). Enzyme inhibitors, cysteine-rich proteins and Nep1-like proteins are located in a plant apoplast and interact with surface receptors and extracellular host targets (Kamoun, 2006; Askani et al., 2021). Another group of cytoplasmic effectors include crinkling and necrosis-inducing families (CRN) that are general for many oomycete members, in a contrast to RXLR that are specific to *Phytophthora* and species of downy mildews (Anderson et al., 2015; Lan et al., 2019). CRN encodes proteins that are characterized by a conserved N-terminal LXLFLAK motif, a recombination site motif HVLVVVP (DWL domain) and diverse C-terminal effector domains (Yin et al., 2017; Xiang et al., 2021).

During the whole genome sequence of the Chinese *P. viticola* isolate JL-7-2, 100 RXLR effectors (PvRXLR) and 90 CRN effectors had been identified; 18 of these PvRXLRs were common with those obtained after the transcriptome sequencing of European *P. viticola* isolate containing 45 RXLR effectors (Mestre et al., 2016; Yin et al., 2017).

In a construction of a cDNA library from zoospores of *P. viticola*, there were 45 expressed sequence tags (EST) potentially encoding hydrolytic enzymes, protein inhibitors, elicitor-like proteins, and RXLR—fungal effectors responsible for its virulence (Mestre et al., 2012). During RNA-Seq analysis of cDNAs in a study of genes involved in a pathogenicity of Australian strain *P. viticola* CSIRO-L-2 and two Chinese strains *P. viticola* JL-7-2 and *P. viticola* ZJ-1-1, total 10 CRN and 51 RxLR (PvRXLR) effectors were identified according to the presence of a secretory signal sequence and an RxLR/CRN motif; all the PvRXLRs were responsible for the suppression of programmed cell death. In addition to that, putative apoplastic effectors were found using Pfam domain analysis; most were identified as glycosyl hydrolases, peptidases, and protease inhibitors (Yin et al., 2015).

The pathosystem between Italian strain of *P. viticola* and *V. vinifera* identified RxLR and CRN genes. In parallel, several RxLR protein effectors were found to be triggering an immune response to the infection of resistant *Vitis riparia* (Brilli et al., 2018). While several effectors of *P. viticola* have been recently studied, little data is available on the function of these molecules in pathogenicity (Li et al., 2015; Yin et al., 2015; Xiang et al., 2016; Brilli et al., 2018). The possible involvement of RxLR effectors on the virulence of specific strains has been further deciphered to elucidate the specific function of these genes in a successful infection on tolerant hosts (Gómez-Zeledón and Spring, 2018). In addition, Brilli et al. (2018) reported that RxLR_PVITv1008311Δsp triggers a hypersensitive response in *V. riparia* but not in *V. vinifera*, suggesting that European-grown grapevine cultivars have lost or perhaps have not yet evolved effector recognition of *P. viticola* to trigger defense responses.

Within the genome sequencing of *P. viticola* isolate INRA-PV221 (Dussert et al., 2019), found 1592 genes that encode proteins concerned with plant-pathogen interactions, while 317 effectors were identified as cytoplasmic RxLRs and several as CRNs. Aside from the two main effector classes, which represent the two dominant classes of secreted effectors, a certain range of other secreted protein families were also found to be encoded in the *P. viticola* genome, namely proteases, glycoside hydrolases, elicitins and elicitin-like proteins, as well as cell wall degrading enzymes, notably pectin lyases, pectin esterases, and phospholipases. Putative carbohydrate-active enzymes CAZymes have also been reported as pathogenicity factors in plant pathogens including oomycetes. Among them, 15 families of glycoside hydrolases, 6 families of carbohydrate esterases, 6 families of carbohydrate binding modules, 4 families of ancillary activities, 3 families of glycosyltransferases, and a polysaccharide lyase (Yin et al., 2017). While the number of CAZymes in the *P. viticola* secretome is lower than in *Phytophthora* species, the glycoside hydrolases family is the most abundant, suggesting that the breakdown of the plant cell wall is an integral step in the successful colonization of the pathogen (Brouwer et al., 2014; Yin et al., 2017). Recently, multiple effector-encoding genes were identified in *P. viticola* transcriptome during the interaction between *V. vinifera* cv. Mgaloblishvili and *P. viticola* (Toffolatti et al., 2020). The latter thus found a possible susceptibility gene, a transcription factor that was down-regulated in the resistant cv. Mgaloblishvili (Toffolatti et al., 2020).

## Life Cycle and Infection Mechanisms

The early phases of plant infection by oomycetes remains not fully understood. Like many oomycetes, *P. viticola* pass through both sexual and asexual stages. There are two phases in asexual reproduction: sporangiogenesis, formation of multinucleate sporangia, and zoosporogenesis, formation of biflagellate zoospore that infects the grapevine host. During pathogen growth, intercellular mycelia reach the substomatal cavity and form a cushion from which sporangiophores arise on the underside of the leaves or stems through stomata and through lenticels in young fruits and form sporangia (under dark conditions with high humidity). Spores are termed sporangia if they germinate indirectly through internal production of biflagellate motile zoospores that are released, encyst and then germinate using a germ tube, or conidia if they germinate directly *via* formation of a germ tube. Spores are generally released by a twisting motion of the sporangiophore during warm, moist conditions. Sporangia discharge their zoospores through the rupture of the sporangium callose apical papilla. Spores stay active from a few hours to days, depending on environmental conditions, and are not able to survive in absence of water since they do not have cell walls. Therefore, mycelium of downy mildew is programmed not to sporulate until humid conditions appear. Yin et al. (2017) have reported that *P. viticola* has lost thiamine biosynthetic pathway encoding genes, similarly to as barley powdery mildew and flax rust. Interestingly, while five oomycetes namely *P. viticola, H. arabidopsidis, P. infestans, A. laibachii*, and *P. sojae*, have lost these thiamine biosynthetic pathway genes, but they conserved the thiamine pyrophosphokinase gene which codes for the phosphorylation of thiamine. Probably because thiamine may be more readily available from the host than other nutrients. Consequently, the thiamine biosynthetic pathway may be considered as the first to be compromised in the course of the evolutionary process toward biotrophy, compared to other metabolic pathways.

### Disease Cycle and Symptoms

*P. viticola* is strictly biotrophic, i.e., it can grow only in association with living cells of its host and not *in vitro*, which makes it difficult to study. Its oospores can land in the soil, where they remain inactive until favorable conditions occur. It will then transfer onto a host plant and start to grow (Agrios, 2005). The life cycle of this pathogen consists of primary and secondary infections (Chen et al., 2019). It overwinters as oospores (resting sexual spores) in leaf litter, shoots, and soil (Rossi et al., 2009) (and, in some cases, as mycelium in infected, but not dead, twigs). In the spring, oospores germinate to produce macrosporangia which, under wet conditions, release zoospores (asexual spores) which encyst and infect when they come in contact with host tissues. The encysted zoospores produce germ tubes which invade the host through stomata and colonize intercellularly the leaf parenchyma with a diploid, non-segmented tubular mycelium that uptakes nutrients from host cells using intracellular haustoria (Allègre et al., 2009).

The period of intercellular mycelium growth is latent with no visible symptoms of infection (Leroy et al., 2013). After 7–10 days, symptoms of primary infection appear on the adaxial side of leaves, typically as yellow areas called “oil spots” that later turn brown due to necrosis as the disease progresses. Under moist conditions and warm temperature (above about 25°C), masses of sporangia emerge through stomata in leaves and lenticels in young fruits at the end of latency (Fröbel and Zyprian, 2019). These sporangia form the “white downy” appearance that will progress to gray. Sporangia produce zoospores that are released through wind or raindrops, starting secondary infections. The ultimate result of leaf injuries is a premature defoliation. In shoots, enlargement of the infected cells and the large volume of mycelium in intercellular areas triggers distortion and hypertrophy, which leads to their death and collapse, generating brown and sunken shoots. After an appearance of sporangiophores in young berries, the intercellular infection causes the breakdown of chlorophyll, then cells collapse and become brown. On adult berries, the fruit's “skin” thickens and its shape distorts. A color change to a reddish brown can be noted as well as a mosaic pattern when grapes are infected. At the end of the growing season, the oomycete forms oospores in the infected leaves and, occasionally, in the shoots and berries. Depending on temperature, humidity, and varietal susceptibility, the full disease cycle takes from 5 to 18 days (Agrios, 2005).

### Proposed Entry Mechanisms

After discharge, zoospores start to move in a search of a new host. The anterior flagellum bends and propagates a sinusoidal wave from its base to a tip, pulling the zoospore forward. The posterior (whiplash) flagellum helps the zoospore to turn. Zoospores detect chemical and electrical gradients (generally non-specific in terms of pathogen-host associations) that attract them to plant surfaces. Once zoospores reach a potential host, they point the flagellum toward the host surface, find a stomatal complex, and encyst. During encystment zoospores detach both flagella, secrete extracellular matrix materials and change ovoid cells to spherical cysts with cellulosic cell walls. Within 30 min of encystment, zoospores germinate using a germ tube that forms an appressorium through the stoma (Allègre et al., 2007). Cyst germination and production of a germ tube return the pathogen to a passive state before restarting reproduction.

**Figure 6:**
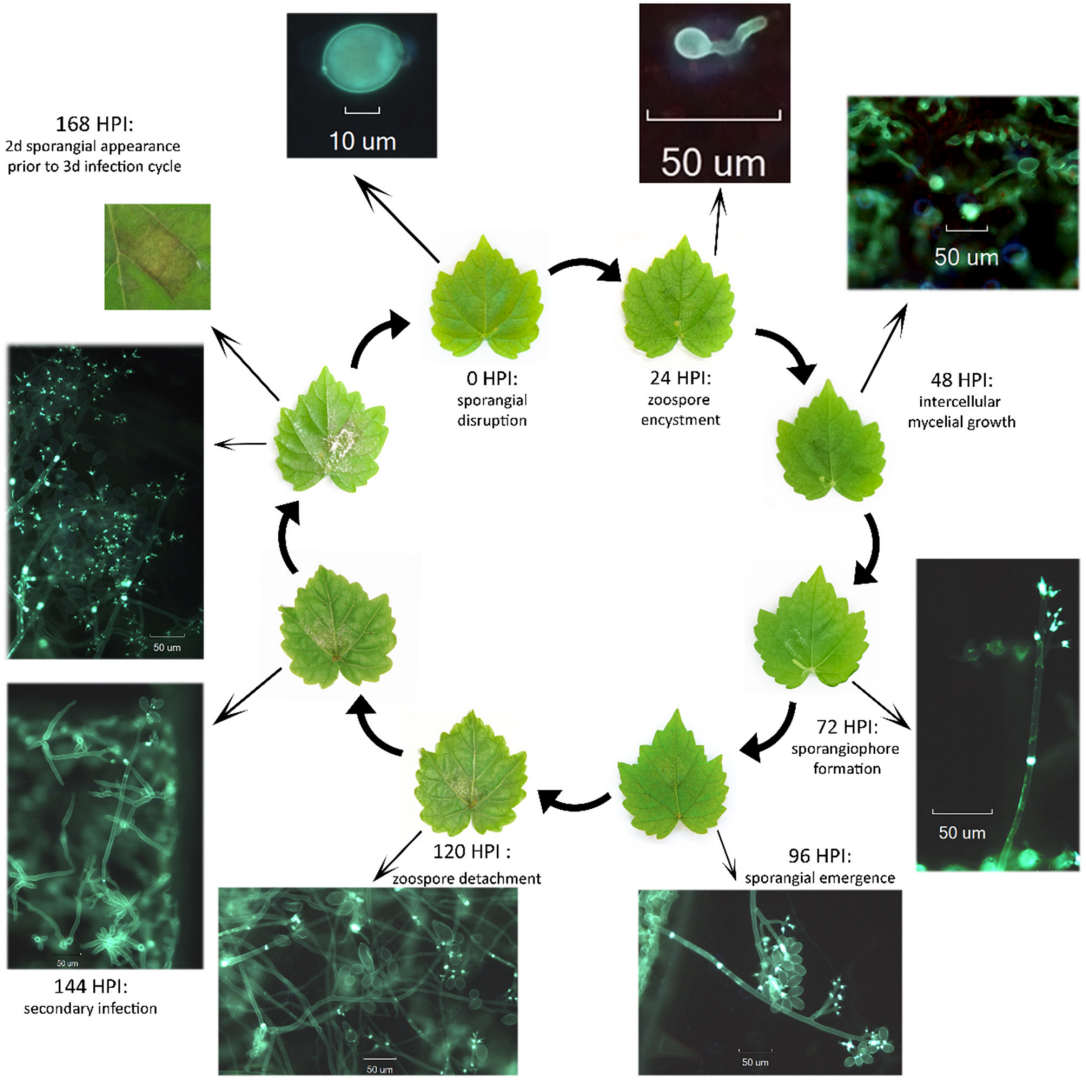
Life cycle of *Plasmopara viticola*. The represented cycle includes asexual stages of the pathogen development, disease cycle and host symptoms connected altogether. The microscopical observations of the pathogen within the plant tissues had been conducted under the epifluorescence microscope (Olympus Bx43, Japan) using a U/B/G filter and images were captured using Infinity Analyze software.

**Figure 7:**
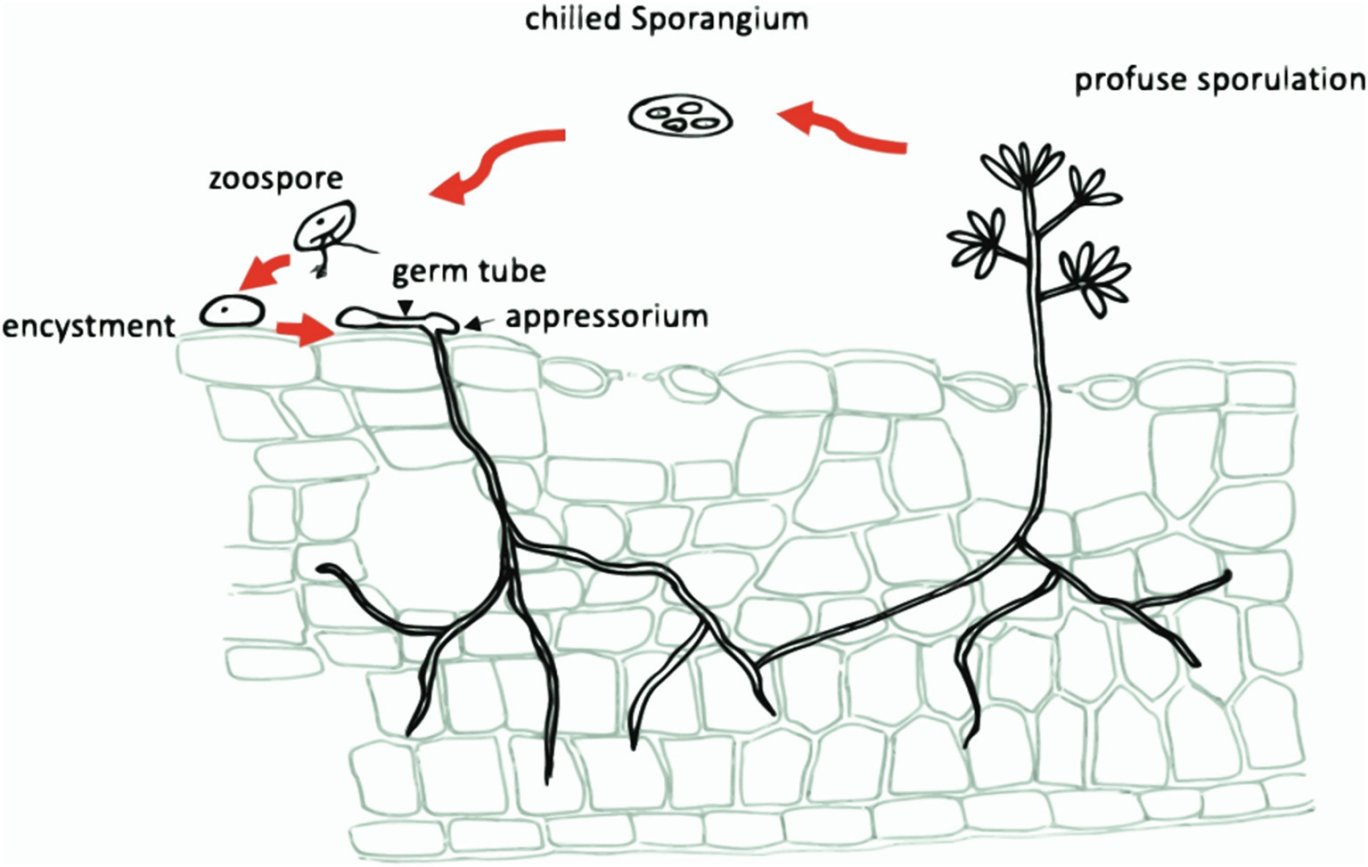
Cross section of leaf infected by downy mildew. The pathogen structures are colored black, and all the stages of asexual life cycle are represented in the same plane of gray-colored plant cells, abaxial surface up.

*P. viticola* hyphae do not remain exterior to the host. Oomycetes produce appressoria that penetrate the plant surface using physical and chemical mechanisms: penetrating by pressure and cell wall-degrading enzymes. A penetration hypha grows from an appressorium underside, passes through the stoma and produces an intercellular, or invasive, hypha. Thin penetration hyphae degrade the plant cell wall, grow through it and invaginate the plasma membrane. This enlarges to form feed structures called haustoria. The invaginated region of a host membrane is called an extrahaustorial membrane. Successful colonization by the pathogen occurs when a susceptible host defense fails and infection culminates in a downy mildew sporulation: sporangia form on the plant surface or sexual spores form inside host tissues, and the life cycle starts again. The asexual cycle of *P. viticola* is schematized in Figures 6, 7 (Agrios, 2005; Tröster, 2016). In addition to asexual reproduction, downy mildews form and propagate oospores. This process provides a genetic variation mechanism and forms spores that can survive for many years in harsh conditions (e.g., lack of water, extreme temperatures or aggressive chemical environments). Normally oospores are produced under rather dry conditions in a huge quantity (up to 250 oospores per mm^2^). Oospores mature during the winter and germinate once exposed to water and a temperature of at least 10°C.

Heterothallic parents—P1 and P2 in *Plasmopara*—form antheridia and oogonia and differ in production and detection of hormones. This establishes a system of hormonal heterothallism, where perception of the opposite hormone establishes compatibility. Once compatibility occurs, gametangia develop and become able to form both selfed and outcrossed couplings. If both hormones are present in heterothallic oomycetes, there is no barrier to selfing, in a contrast to true fungi where sexual dimorphism or self-incompatibility inseparably linked with a heterothallism (Agrios, 1969; Wong et al., 2001).

During sexual reproduction, parental gametangia undergo meiosis to produce a haploid nucleus. Small male antheridia grow on the surface of large female oogonia (unfertilized eggs) and produce fertilization tubes which penetrate the oogonium. The single antheridial haploid nucleus passes through a fertilization tube into the oosphere and fuses with a single haploid nucleus from the oosphere, forming a diploid oospore (fertilized egg cell or zygote). Oogonia contain about 10 gametic nuclei, but only 1 oogonic nucleus fertilizes successfully, giving birth to the diploid zygotic nucleus. The remaining unfertilized oosphere nuclei migrate to the periplasmic space and degenerate, surrounded by a thick double-layered oogonium wall with inner and outer oosphere walls. Oospores mature when the previously thin inner cell wall becomes thick and then a dormancy period occurs, during which the oospore does not germinate even under optimal conditions. Subsequent germination depends on several important factors such as light, temperature, humidity, plant extracts and microbial metabolites. Germinated oospores form primary zoosporangia (macrosporangia), which release zoospores that act as described above (Burruano, 2000; Agrios, 2005; Lamour and Kamoun, 2009; Spencer-Phillips et al., 2016; Tröster, 2016).

## How Grapevine Plants React

### Perception of the Pathogen

During contact between pathogens and host plants a dynamic evolutionary battle rise that can be explained as a “zigzag” model (Jones and Dangl, 2006). To prevent infection by biotrophic pathogens, including the downy mildew, plants have evolved two complex signaling systems that sense pathogens or disease-related disorders. In addition to detecting pathogen molecular patterns, they are also able to sense the pressure produced by hyphae, which try to breach the plant's epidermis. During early stages of the infection, plant responses to oomycetes are identical, just less effective and slower in susceptible plants (Silvar et al., 2008; Huibers et al., 2009). Plants recognize wide conserved pathogen-associated molecular patterns (PAMPs) through pattern recognition receptors (PRRs) situated in plasma membranes, thus triggering the first barrier of innate immune system called PAMP-triggered immunity (PTI) (Jones and Dangl, 2006). Some pathogens developed a virulence through which they avoid or suppresses resistance reactions by secreting effector proteins that suppress the PTI signaling pathway, as well as modulating the host metabolism to enable the biotrophic interaction. In a response, plants developed an effector-triggered immunity (ETI), which involves the perception of specific effectors either directly or indirectly by R proteins. Once effectors are recognized, the plant will activate a second circle of defense reactions including hypersensitive response (HR) and systemic acquired resistance (SAR) (Brader et al., 2017; Esmaeel et al., 2018).

In broad terms, the compatible interaction between grapevines and *P. viticola* is marked by an overall down-regulation of photosynthesis-related processes and an impaired up-regulation of genes encoding pathogenesis-related (PR) proteins, enzymes of the phenylpropanoid pathways, and stimulus response regulators (Nascimento et al., 2019). In response to *P. viticola*, several infection genes are triggered including the stomatal movement related, BLUS1 (Takemiya et al., 2013), a growth related gene, bZIP11 (Weiste et al., 2017), a detoxification-related gene, the glutathione peroxidase gene, and an abiotic stress regulation gene ERF38 (Fu et al., 2020).

### Host Resistance to Downy Mildew

Throughout their life, plants are exposed to a wide range of potential pathogens and pests. Therefore, they developed complex mechanisms to protect themselves from diseases. Normally defense mechanisms are triggered after the pathogen attack, leading to induced resistance. Depending on the inducer, resistance can be defined as either SAR and induced systemic resistance (ISR) (Pieterse et al., 1996). Besides inducible defense response, plants use preformed resistance including structural and biochemical factors. Structural aspects (e.g., properties of the leaf surface or resistant cell walls) are the first barrier that pathogens face before invading the plant. If the microorganism overcomes this line of non-specific resistance and colonizes the plant successfully, the plant becomes its host and induced resistance mechanisms then respond (Slusarenko et al., 2000; Mysore and Ryu, 2004).

Phytohormones play a crucial role as signaling molecules for disease resistance. Perception of oomycete pathogen by the plant triggers a series of signaling physiological and metabolic pathways in cells by regulating resistance-related genes and initiating the corresponding immune responses including HR (Koornneef and Pieterse, 2008; Halim et al., 2009). Effective defense against biotrophic pathogens is considered to depend largely on triggering the defense responses controlled by the SAR-dependent pathway, leading to host's programmed cell death (Glazebrook, 2005).

Plant defense responses require energy and activation of signaling molecules, supplied by primary metabolism of carbohydrates, organic acids, amines, amino acids, and lipids. Immune responses include the formation of salicylic acid (SA), jasmonic acid (JA), ethylene and abscisic acid (ABA) (Bari and Jones, 2008). Some beneficial microorganisms are able to not only directly inhibit pathogenic growth *via* hyperparasitism or metabolic activities and supply plant by nutrients or hormones, but also they have an ability to stimulate plant defense mechanisms, which lead to ISR (Pieterse et al., 1996; Van Loon et al., 1998). ISR is characterized by rapid molecular and cellular defense responses, priming the plant for more fast and effective reaction to pathogen infections, controlling plant energy costs. These responses differ and depend on the plant/pathogen/beneficial agent system.

Induction of systemic resistance occurs when host-specific receptors recognize pathogenic or beneficial microorganisms through pathogen-molecular patterns (PAMPs) or microbe-associated molecular patterns (MAMPs), elicitors that are produced by invasive microorganisms to suppress plant defense and include peptides, metabolites, cell wall components, enzymes, and toxins (Jones and Dangl, 2006). Damaged host cells produce damage-associated molecular patterns (DAMP), recognized by the pattern recognition receptors (PRRs) that trigger specific cell signals, leading to PAMP-triggered immunity (PTI). These cell signals induce an expression of defense genes, coding the synthesis of antimicrobial compounds and defense proteins involved in the management of oxidative stress, pathogen degradation, compounds mobilized to strengthen the host cell walls, and change plant metabolism toward defense (Kushalappa et al., 2016; Savini et al., 2018; Héloir et al., 2019).

*P. viticola* is known to trigger several defense responses in leaf tissues, including physical (i.g. lignin, deposition callose) and chemical (i.g. synthesis of chitinases, glucanases, peroxidases, stilbenes) barriers, and factors that are involved in lignification processes as well as necrosis that may lead to resistance development (Dai et al., 1995). For instance, peroxidase was reported as age-related resistance in a number of grapevines-pathogen interactions including *P. viticola* (Reuveni, 1998; Steimetz et al., 2012).

HR is a generation and accumulation of reactive oxygen species (ROS), which play a crucial role in resistance to pathogens either by directly reinforcing plant cell walls trough cross-linking of glycoproteins, lipid peroxidation or triggering the ROS-signaling networks resulting to the establishment of a local cell death around the point of infection, which blocks further development of biotrophic parasites (Govrin and Levine, 2000; Hernández et al., 2016). Local cell death is a type of programmed cell death (PCD), mechanism that is specific for all organisms from bacteria to multicellular eukaryotes (Locato and De Gara, 2018).

Systemic acquired resistance is characterized by a diffusion of hormonal signals from the invasive point through the whole plant, informing cells of all the organs, both infected and not, to activate their defenses for further attack, and launching a wide-spectrum resistance in tissues distant from the site of initial infection. SAR is usually induced after the exposure to abiotic or biotic elicitors, related to localized necrotic lesions. It is associated with an accumulation of the phytohormone SA, playing an important role in signal transfer, and activation of defense-related genes, encoding pathogenesis-related (PR) proteins with antimicrobial activity (Corina Vlot et al., 2009). An upregulation of genes involved in the biosynthesis of JA and an increase of JA levels suggest the crucial role of this hormone in *V. riparia* resistance toward *P. viticola* (Polesani et al., 2010). The up-regulation in *VvWRKY1* overexpressors related to JA metabolism and signaling has been demonstrated to increase tolerance to fungal pathogens (Marchive et al., 2013).

Structural mechanisms, aimed to produce physical barriers against infection, include deposition of cutin, cuticular waxes, lignin and callose for cell wall reinforcement at infection sites (O'Connell and Panstruga, 2006; Miotto-Vilanova et al., 2016; Paris et al., 2016; Ali et al., 2018; El-Sharkawy et al., 2018). Cell wall deposition or papillae formation might result from PTI that is mediated by receptor-like kinases (RLKs) or receptor-like proteins (RLPs) localized in plant cell membrane (Boutrot and Zipfel, 2017).

Antimicrobial secondary metabolites like phytoanticipins (mainly saponins) and phytoalexins form a biochemical resistance. The main stilbenic phytoalexins in grapevine are resveratrol and its derivatives: viniferin, glycosylated piceid and pterostilbene. Stilbenic phytoalexins showed biological activity against several grapevine pathogens and are considered as markers of disease resistance (Coutos-Thévenot et al., 2001; Pezet et al., 2003; Aziz et al., 2006, 2016). Resistant grapevine cultivars react quickly to *P. viticola* attacks by producing high levels of stilbenes at the infection area, and have significant inhibitory impacts on the motility of *P. viticola* zoospores and therefore subsequent disease development (Pezet et al., 2004). These findings endorse the fundamental role and efficiency of phytoalexins in the grapevine resistance mechanisms to downy mildew (Alonso-Villaverde et al., 2011).

### What Makes Plants Resistant or Susceptible?

Plants react to both biotic and abiotic factors to activate of set of genes that encode stress-related proteins for signaling, sensing, and defending the host against changes. The vulnerability of *Vitis vinifera* to downy mildew implies that this species does not have a specific identification system for *P. viticola* that would allow the triggering of an efficient defense mechanism (Di Gaspero et al., 2007). Transcriptional changes related to *P. viticola* infection of susceptible grapevines have been linked to a weak defense response (Polesani et al., 2010) and to an establishment of a compatible interaction (Liu et al., 2016). On the other hand, resistant genotypes exhibit a strong and fast transcriptional reprogramming of processes connected to defense, signal pathway, and a secondary metabolism (Kortekamp, 2006; Figueiredo et al., 2012). These changes include an acceleration of the expression of genes encoding PR proteins and phenylpropanoid biosynthesis, and those involved in the modulation of signal transduction components and markers of hypersensitive response in resistant grapevine cultivars (Malacarne et al., 2011; Figueiredo et al., 2012). These data imply that tolerant and susceptible cultivars not only have a separate metabolic pattern, but also react quite differently when challenged with biotic stress. Within plant-pathogen interactions, pathogens and their elicitors that are governed by AVR genes confront pathogenesis-related proteins (PR) and resistance (R) metabolites, regulated by related genes. So, disease resistance is a quantitative characteristic, which is regulated by complex of genes. To investigate differences between susceptible and resistant strains and select the most appropriate plant genotypes, analysis of R-gene expression levels had been used since the development of genomic sequencing, in addition to trait-specific germplasm identification, quantitative trait loci (QTLs) mapping and other genetic approaches (Zhao et al., 2019).

## Disease Management

### Breeding Approaches

The best and most sustainable tool in crop protection is the breeding of resistant grapevine cultivars. Unfortunately, the successful introgression of resistance loci inside of existing cultivars in conventional breeding techniques typically takes ~15 years and is expensive. Within crops, important traits representing productivity, stress tolerance and disease resistance are complex, polygenic, and quantitative, which means that numerous genes control genetic variation of these traits in each culture. Identification and genome mapping of QTLs, DNA sections correlating with variation of quantitative traits, is carried out using molecular markers. DNA-markers are used in gene incorporation in cultivars selected for introduction. With methods of map-based tagged genes cloning, constituting marker-assisted selection (MAS), QTL mapping allows us to understand an association between plant genotype and phenotype and use this strategy for the creation of new cultivars with optimal agronomic traits, including disease resistance (Keller et al., 2000).

The development of genome editing approaches, such as well-known CRISPR-Cas9, let scientists to change gene structure and expression in a targeted way for many purposes including disease resistance. This technology was adapted from a naturally occurring genome editing system that bacteria use as an immune defense to “remember” bacterial viruses and cut the viral DNA in subsequent attack which disables the virus. Once introduced into cells, the guide RNA cuts the DNA at the targeted location (usually using Cas9 enzyme) and then researchers use the cell's own DNA repair machinery to add or delete pieces of genetic material, or to make changes to the DNA by replacing an existing segment with a customized DNA sequence (Gupta and Musunuru, 2014; Wang et al., 2016).

While the majority of traditional European cultivars (*Vitis vinifera*) are mildew-susceptible, some American (*V. rotundifolia, V. rupestris, V. labrusca, V. riparia, V. cinerea*) and Asian (*V. amurensis, V. piasezkii, V. coignetiae*) grapevine cultivars exhibit partial to total resistance to downy mildew, so their germplasms are still the main source of genetic resistance to *P. viticola* (Jackson, 2000; Cadle-Davidson, 2008; Díez-Navajas et al., 2008). In recent decades, genetic analysis of a resistance to downy mildew resulted in identification of the most important genetic resistance factors with their corresponding loci (Schneider et al., 2019). Since the genetic identification of the first resistance locus against downy mildew, resistance to *P. viticola* (*Rpv*) 1 in *Muscadinia rotundifolia* (Merdinoglu et al., 2003), 31 *P. viticola*-resistance loci have been mapped in American and Asian wild accessions exploiting first- and second-generation molecular markers. Using backcrosses between parental lines of susceptible *V. vinifera* obtaining the best agronomic parameters (yield, growth, organoleptics) and genotypes carrying different *Rpv* loci, multiple studies on varietal selection were carried out.

Among the *Rpv* mapped in *Vitis* and *Muscadinia*, five (*Rpv8, Rpv10, Rpv12, Rpv25*, and *Rpv26*) were derived from the East Asian species *V. amurensis* (Fu et al., 2020). The locus *Rpv1* was introduced from *V. rotundifolia*, located on its chromosome 12 and used in selection of such cultivars as Artaban, Floreal, Voltis (Merdinoglu et al., 2003; Savini et al., 2018; Schneider et al., 2019). *Rpv2* from the chromosome 18 of *V. rotundifolia* was responsible for total resistance of its progenies (Gessler et al., 2011; Merdinoglu et al., 2018). Locus *Rpv3*, mapped on chromosome 18 and originated from American *V. rupestris*, is characterized by multiple resistance alleles (Bellin et al., 2009; Schwander et al., 2012; Schneider et al., 2019). Popular resistance haplotype *Rpv3-1* was maintained in such cultivars as Regent, Bianca, Villard blanc, Cabernet blanc, Kozma 20-3 (Bellin et al., 2009; Ciubotaru et al., 2021; Wingerter et al., 2021). *Rpv3-3* was found in Seyval, Merzling and Solaris grapevine. *Rpv4*, originated from American grapevine, was identified in Regent. Introduced from *V. riparia, Rpv5* was found on a chromosome 9 in a progeny between Cabernet Sauvignon and Gloire de Montpellier and *Rpv6* was identified in the same research with the only distinction that it was mapped on chromosome 12 (Marguerit et al., 2009; Schwander et al., 2012). Locus *Rpv8* from *V. amurensis* was found to confer total resistance and belong to chromosome 14 (Blasi et al., 2011). *Rpv10* originated from *V. amurensis* was mapped on chromosome 9 in Solaris and was introduced in cultivars Bronner, Divico, and Divona (Schwander et al., 2012; Schneider et al., 2019). *Rpv11*, founded on chromosome 5 in a population from Regent, was related to low level of downy mildew resistance in some cultivars of Regent, Chardonnay and Solaris (Schwander et al., 2012; Van Heerden et al., 2014). Introduced from *V. amurensis*, dominant *Rpv12* was detected in a chromosome 14 of Kozma 20-3 cultivar together with *Rpv3-1* (Venuti et al., 2013; Kim et al., 2017; Possamai et al., 2020). Recently, linkage analysis identified three QTLs for *P. viticola* resistance: *Rpv22* on LG 02, *Rpv23* on LG15, and *Rpv24* on LG18 (Fu et al., 2020). *P. viticola* trigger the expression of the NBS-LRR-like gene RPP8, and might be considered as a candidate gene for Rpv23 (Fu et al., 2020). Additional class of R gene, LRR receptor-like serine/threonine-protein kinase (LRR-RLK), which have been characterized in the Rpv24 region, denoting that these two genes could also play a role in disease resistance mechanisms. Two stable QTLs had been located on LG 15 in a study of progeny derived from the cross between *V. vinifera* L. cv. Red Globe and *V. amurensis* Rupr. cv. Shuangyou: Rpv25 and Rpv26 (Lin et al., 2019). A QTL named Rpv27 was detected on a chromosome 18 during a mapping population study of a cross between *V. aestivalis* hybrid Norton and *V. vinifera* cv. Cabernet Sauvignon (Sapkota et al., 2019). A locus named Rpv28 had been found on a chromosome 10 during a study of the population derived from a cross between *V. rupestris* cv. Scheele and *V. riparia* Michx (Bhattarai et al., 2021). In a population mapping study of Georgian-derived accessions (Southern Caucasus), 3 novel loci Rpv29 (Chr14), Rpv30 (Chr3), and Rpv31 (Chr16) had been discovered (Sargolzaei et al., 2020).

### Chemical Control

Despite cultural practices and the selection of resistant cultivars, currently, no effective biological control measures are known against *P. viticola*. Consequently, chemical control remains the most effective and economical strategy to protect crops from downy mildews under conditions favorable to disease development. Copper-based fungicides, the oldest products in plant protection, are still used in viticulture, in spite of long-term accumulation of Cu in soils, leading to anthropogenic pollution, negatively affecting soil fertility and microbiota (Pietrzak and McPhail, 2004; Thuerig et al., 2018a). In recent years, use of copper was reduced due to development of new fungicide formulations and safer application techniques; nevertheless, reduction or elimination of crop copper and synthetic pesticides is one of the most important aims for researchers in plant protection.

In spite of high level of protection against harmful plant diseases, chemical control faces some challenges. Firstly, oomycetes penetrate into the leaf tissues by formation of hyphae and haustoria (Toffolatti et al., 2011a). Many fungicides, especially contact types, accumulate on and in the waxy cuticle layer and do not penetrate deeper into the leaf (Vladimíra Zelená, 2007). Also, these pathogens can overwinter in soil and fallen leaves for long periods (Toffolatti et al., 2011b), while pesticides require multiple applications (Halleen et al., 2016). In addition the problem of potential harm to humans and the environment (Chitarrini et al., 2017), development of acquired fungicide resistance and emergence of new pesticide-resistant pathogenic strains are ongoing concerns (Leroux et al., 1999; Lucas et al., 2015; Campbell et al., 2021; Massi et al., 2021).

The most widely used technique to control downy mildew in vineyards, including organic ones, are multiple applications of copper compounds such as oxychloride, hydroxide, sulfate oxide (Tromp and De Klerk, 1988; Hofmann and Kauer, 2008; La Torre et al., 2008; Rusjan, 2012). Bordeaux mixture, a water solution of copper sulfate (CuSO_4_) and lime [Ca(OH)_2_], has been used since 1882, when French botanist A. Millardet found an inhibitory effect of copper against downy mildew (Millardet, 1885; Vecchione et al., 2007; Gessler et al., 2011; Zhang et al., 2017). Burgundy mixture, a water solution of copper sulfate and sodium carbonate, has been used since 1887 (Masson, 1887; Gessler et al., 2011; Rusjan, 2012).

Copper-based compounds are included in “protectant” multi-site fungicides that are used in pre-infection applications to form a protective barrier at the plant surface. This group includes dithiocarbamates such as mancozeb, zineb, maneb, propamocarb, metiram, propineb (Clifford and Bruyns-Haylett, 1978; Falk et al., 1996; Gisi, 2002; Šrobárová et al., 2007; Gisi and Sierotzki, 2008; Patil, 2015); phthalimide group, represented by captan, captafol, and folpet (O'Neill et al., 1996; Madden et al., 2000; Genet et al., 2006; Rekanovic et al., 2008; Gullino et al., 2010; Jermini et al., 2010a; Gessler et al., 2011; Barros et al., 2018); phthalonitrile chlorothalonil (Northover and Ripley, 1980; Gisi et al., 2007; Lebeda et al., 2008; Barros et al., 2018) and quinone dithianon (Corio-Costet, 2011; Nanni et al., 2016). Phenylamides and aluminum are two of the most broadly applied systemic fungicides to manage *P. viticola*.

Single-site fungicides provide both pre- and post-infection protection and include four main chemical classes: phenylamide compounds such as mefenoxam, metalaxyl, and benalaxyl (Magarey et al., 1991; Nicholas et al., 1994; Falk et al., 1996; Underdown et al., 2008); strobilurines such as azoxystrobin, trifloxystrobin, famoxadone, and fenamidone (Reuveni, 2001; Wong and Wilcox, 2001; Genet et al., 2006); the carboxylic acid amides like dimethomorph, iprovalicarb, flumorph, benthiavalicarb, mandipropamid (Wicks and Hall, 1990; Albert et al., 1991; Gisi et al., 2007; Reuveni, 2014; Nanni et al., 2016; Kim et al., 2017); and cyano-acetamide-based oxime carbonates like cymoxanil (Gullino et al., 1997; Genet and Vincent, 1999; Joshi, 2003). Both cymoxanil and chlorothalonil are specific, non-systemic, mildew fungicides and are curative if they are applied during the first few days post infection. Less popular single-site fungicides include phosphonates as fosetyl-Al (Magarey et al., 1991; Gisi, 2002; Rekanovic et al., 2008) and dinitroaniline fluazinam (Gisi and Sierotzki, 2008; Lebeda et al., 2008; Espinel-Ingroff, 2009).

### Organic Disease Management

Crop protection agents suitable for organic agriculture include plant, animal, microbial and mineral products, and possess different modes of action: antibiosis, induction of resistance and hyperparasitism (Dagostin et al., 2011). Several products, based on inorganic materials are currently registered and applied in plant protection against grapevine downy mildew.

Potassium hydrogen carbonate showed inhibitory activity against spores of *P. viticola* and high level of protection in field applications under moderate to low infection levels (La Torre et al., 2008, 2019; Parveaud et al., 2010; Dagostin et al., 2011) and in greenhouse (Lukas et al., 2016). Potassium phosphonate was effective against downy mildew in field conditions, but its application leads to phosphonate residues in wine; the level of accumulation depends on the application strategy (Speiser et al., 2000; Vecchione et al., 2007; Hofmann and Kauer, 2008; Parveaud et al., 2010; Corio-Costet, 2011; Ball et al., 2014). Lime sulfur, a mixture of calcium polysulfides and thiosulfate, was effective under controlled conditions (Lukas et al., 2016). Propolis was tested in semi-controlled conditions and showed promise for suppressing pathogen growth (Dagostin et al., 2011). Oligochitosan, a deacetylated derivative of chitin, was found to promote plant defense reactions. It increased accumulation of phytoalexins, resveratrol and ε-viniferin, significantly reducing infection severity on grapevine under natural and semi-controlled conditions (Aziz et al., 2006; Vecchione et al., 2007; Dagostin et al., 2011; Romanazzi et al., 2014).

Among the synthetic materials, beta-amino-butyric acid (BABA), benzothiadiazole (BTH) and high rates of Tween 80 (250 ml/l) were effective under controlled conditions. Only BTH is registered as a commercial product, Bion (Dagostin et al., 2011; Savini et al., 2018). The application of non-protein amino acid BABA (DL-3-amino-n-butanoic acid, beta-aminobutyric acid) efficiently protected field-grown grape against *P. viticola*. BABA decreased sporulation when applied on leaf discs (Cohen et al., 1999; Vecchione et al., 2007; Kim Khiook et al., 2013), on detached leaves (Dubreuil-Maurizi et al., 2010), in potted plants when the root system was treated (Cohen et al., 1999), and in a greenhouse (Slaughter et al., 2008; Dagostin et al., 2011) and in field trials (Reuveni et al., 2001). BTH is a synthetic (and less phytotoxic) functional analog of salicylic acid and is registered in the EU as a crop resistance inducer. Results of test on leaf disks showed that sporal growth was inhibited up to 100% at high concentration (Cohen et al., 1999; Dufour and Corio-Costet, 2012). On the other hand, there was no direct toxic effect against sporangia germination after treatments with BTH on detached leaves. In the same study under greenhouse conditions, leaf treatments decreased disease severity even when applied 14 days before pathogen inoculation (Perazzolli et al., 2008; Burdziej et al., 2021).

### Plant Extracts

Preparations obtained by the extracting of a specific solvent on a dried or fresh plant, including plant derivates (for example saponins) and oils sometimes show high level of effectiveness under controlled conditions but little success in the field. This is generally related to their high water solubility (Chapagain and Wiesman, 2006). Despite a wide range of protective properties of plant-derived products for plant protection, its usage in commercial agriculture is limited by high cost, low persistence and low-level of rainfastness.

In studies on leaf discs, extracts of *Artemisia absinthium, Artemisia vulgaris, Achillea millefolium, Betula pendula, Calendula officinalis, Cannabis sativa, Chloris virgata, Daucus carota, Dalbergia hupeana, Dryopteris filix-mas, Frangula alnus, Glycyrrhiza glabra, Hibiscus trionum, Hypericum perforatum, Hyssopus officinalis, Magnolia officinalis, Origanum vulgare, Paeonia suffruticosa, Pastinaca sativa, Petasites albus, Picea abies, Pinus massoniana, Pinus pinaster, Rheum palmatum, Robinia pseudoacacia, Salix alba, Salvia officinalis, Symphytum officinale, Solidago canadensis, Tanacetum vulgare, Trigonella foenum-graecum*, and *Traxacum officinalae* showed high inhibitory effect on sporangia germination (Kast, 2001; Chen et al., 2002; Godard et al., 2009; Harm et al., 2011; Tröster, 2016; Gabaston et al., 2017a,b; Andreu et al., 2018; James, 2018; Thuerig et al., 2018b).

Under field and greenhouse conditions, extracts of *Equisetum arvense, Frangula alnus, Glycyrrhiza glabra, Inula viscosa, Larix decidua, Quillaja saponaria*, and *Yucca schidigera* controlled grapevine downy mildew (Bennett and Lynch, 1981; Cohen et al., 2006; Dagostin et al., 2011; Arnault et al., 2012; Thuerig et al., 2016, 2018; Mulholland et al., 2017; James, 2018; La Torre et al., 2019). Extracts of *Abies sibirica, Juncus effusus, Magnolia officinalis, Rheum rhabarbarum* L., *Rheum palmatum, Salvia officinalis*, and *Solidago virgaurea* were able to suppress pathogen growth only under semi-controlled conditions (Kast, 2001; Godard et al., 2009; Dagostin et al., 2010, 2011; Harm et al., 2011; Toffolatti et al., 2011a; Thuerig et al., 2016; James, 2018; Ramseyer, 2018; La Torre et al., 2019).

Plant essential oils of *Cymbopogon citratus, Cinnamomum zeylanicum, Eucalyptus globulus, Melaleuca alternifolia, Mentha piperita, Origanum majorana, Origanum vulgare, Thymbra spicata* and citrus were effective under controlled conditions, but most studies showed decreased inhibitory activity in the field, especially with a long incubation period (Dagostin et al., 2010, 2011; La Torre et al., 2014; Maia et al., 2014; De Oliveira Fialho et al., 2016; Rienth Id et al., 2019).

Several plant derivatives significantly reduced disease expression under semi-controlled conditions. Fatty acid-based product Tecnobiol, containing oleic and linoleic acids, showed a good preventive action in greenhouse conditions but and less effectiveness in the field, where it requires serial applications (Palla, 2006; Dagostin et al., 2011).

Lecithin, a mixture of glycerophospholipids extracted from *Glycine max*, is applied against powdery mildew of different crops (Ramseyer, 2018). This substance showed high fungitoxic effect on sporangial germination *in vitro* and middle level of protection in field trials (Aveline and Chovelon, 2016; Marchand, 2018). Extract of the brown alga *Ascophyllum nodosum* reduced disease expression in laboratory and greenhouse experiments (Lizzi et al., 1998; Dagostin et al., 2011). Also, the brown alga *L. digitata* has been observed to trigger the grapevine plant defense mechanism and protect them against pathogens including *P. viticola* (Aziz et al., 2003).

The β-1,3-glucan laminarin derived from the brown alga *Laminaria digitate* is authorized for organic farming in EU (Ball et al., 2014). It effectively reduced *P. viticola* damages by plant defense responses in leaf disk assay (Gauthier et al., 2014), under semi-controlled conditions (Aziz et al., 2003; Trouvelot et al., 2008; Allègre et al., 2009; Romanazzi et al., 2014) and in the field when applied with a surfactant (Paris et al., 2016). The elicitor primed H_2_O_2_ production at the infection area, triggers the expression of defense genes, callose, and phenol depositions, and HR-like cell death (Trouvelot et al., 2008).

### Biological Control

Like many other plants, grapevine can be colonized by huge numbers of microbial organisms in exterior (epiphytes) and interior (endophytes) surfaces of aboveground tissues and in the rhizosphere (Pinto et al., 2014; Zarraonaindia et al., 2015; Asghari et al., 2019). Biological control encompasses the use of beneficial microorganisms, such as specialized fungi and bacteria to assault plant pathogens and control diseases they cause (Table 3). Biological control offers an environmentally friendly approach to handle plant diseases as part of an integrated pest management system. In the EU Pesticide Database, 65 strains of microorganisms are currently approved in plant protection: 36 strains of fungi, yeasts, and oomycetes; 21 strains of bacteria; and 7 viruses (ec.europa.eu, actual data for June 2020). Among these agents, 32 strains possess fungicidal properties and 15 of them can be used against oomycetes.

**Table 3:** Microorganisms based pesticides used against downy mildews.

**Biocontrol strategy**	**Species used**	**Possible mode of action**	**References**
Bacteria	*Bacillus amyloliquefaciens* FZB24	Hyperparasitism and induced resistance	Savini et al., 2018
	*Bacillus amyloliquefaciens* QST 713	Induced resistance	Savini et al., 2018
	*Bacillus subtilis* GLB191	Antifungal metabolites	Zhang et al., 2017
	*Bacillus subtilis KS1*	Antifungal lipopeptide iturin A	Furuya et al., 2011
	*Bacillus pumilus* QST 2808	Hyperparasitism and antifungal metabolites	Savini et al., 2018
	*Bacillus pumilus* GLB197	Antifungal metabolites	Zhang et al., 2017
	*Bacillus velezensis* KOF112	Zoospore release inhibition and induced resistance	Hamaoka et al., 2021
	*Lysobacter capsici* AZ78	Cell wall degradation due to lytic enzymes and anti-germinative metabolite diketopiperazine	Puopolo et al., 2014b; Cimmino et al., 2020
	*Ochrobactrum* sp. SY286	Cell wall degradation	Zang et al., 2020
	*Paenibacillus* sp. B2	Lytic enzymes (paenimyxin) and induced resistance	Hao et al., 2017
	*Pseudomonas fluorescens* PTA-CT2	Induced resistance	Lakkis et al., 2019
	*Pseudomonas fluorescens* Pfl	Induced resistance	Archana et al., 2011
	*Streptomyces atratus* PY-1	Zoosporicidal activity due to metabolite production	Liang et al., 2016
	*Streptomyces* sp. ANK313	Zoosporicidal activity due to metabolite khatmiamycin	Abdalla et al., 2011; Yoshioka et al., 2019
	*Streptomyces viridosporus* HH1	Induced resistance	El-Sharkawy et al., 2018
	*Streptomyces violatus* HH5	Induced resistance	El-Sharkawy et al., 2018
Yeasts	*Aureobasidium pullulans*	Induced resistance	Harm et al., 2011
Fungi	*Acremonium persicinum*	Anti-germinative metabolites acremines	Lo Piccolo et al., 2015
	*Acremonium sclerotigenum*	Anti-germinative metabolites acremines	Assante et al., 2005; Burruano et al., 2008
	*Alternaria alternata*	Antifungal metabolites diketopiperazines	Musetti et al., 2006
	*Epicoccum nigrum*	Hyperparasitism	Kortekamp, 1997
	*Fusarium delphinoides*	Hyperparasitism and lytic enzymes	Ghule and Sawant, 2017
	*Fusarium brachygibbosum*	Hyperparasitism and lytic enzymes	Ghule and Sawant, 2017
	*Fusarium pseudonygamai*	Hyperparasitism and lytic enzymes	Ghule and Sawant, 2017
	*Fusarium* sp. MCC 134	Hyperparasitism and lytic enzymes	Ghule and Sawant, 2017
	*Fusarium proliferatum*	Cell wall degradation	Falk et al., 1996; Bakshi et al., 2001
	*Penicillium chrysogenum*	Induced resistance	Thuerig et al., 2006
	*Phomopsis* sp. CAFT69	Zoosporicidal activity due to phomopsidin metabolites	Talontsi et al., 2012
	*Rhizophagus irregularis*	Induced resistance	Bruisson et al., 2016; Cruz-Silva et al., 2021
	*Trichoderma harzianum* T39	Induced resistance due to VOCs	Perazzolli et al., 2011; Lazazzara et al., 2021
	*Trichoderma harzianum* HL1	Induced resistance	El-Sharkawy et al., 2018
	*Trichoderma viride* HL5	Induced resistance	El-Sharkawy et al., 2018

The most popular formulation used at market is a wettable powder (WP) consisting of the lyophilized BCA cells, spores or conidia combined with wetting agents and sometimes bulking agents. Wettable powders are supplied in measured sachets to simplify premixing and reduce the contact between the product and environment or operator. Once solubilized, biocontrol products can be applied as a dilute suspension through liquid spraying equipment and drip type irrigation systems. Suspension concentrate (SC) is a liquid form of biocontrol products, more popular for bacterial agents. Less popular (nowadays) dry biocontrol formulations include dusts (DP) and powders for seed dressing (DS) when there is a need of protection against soil-born pathogens; granules (GR), micro granules (MG) and water dispersible granules (WG). Liquid formulations include emulsions, oil dispersions (OD), suspo-emulsions (SE), capsule suspensions (CS) and ultra low volume formulations (Gasic and Tanovic, 2013).

#### Fungal Agents

Over the last decades, several microorganisms isolated from different plant organs have been screened as BCAs to control *P. viticola* (Table 3). Endophytic fungi *Acremonium persicinum* and *Acremonium sclerotigenum* (the latter incorrectly identified as *Acremonium byssoides*), isolated from asymptomatic grapevine, showed a hyperparasitism on *P. viticola*. Culture filtrates, crude extracts and secondary metabolites acremines inhibited sporangial germination of *P. viticola* in anti-germinative assays (Assante et al., 2005; Burruano et al., 2008; Lo Piccolo et al., 2015). In a study on leaf disks, asexual reproduction of the pathogen was significantly inhibited: sporangiophores were found thin and deformed, growing only at the points of inoculation. In leaves, colonized by this endophyte, formation of sexual structures (gametangia and oospores) of the pathogen was earlier than in non-inoculated leaves, apparently an attempt to ensure its survival in a response to stress (Burruano et al., 2008).

*Aureobasidium pullulans* was tested outdoors (in a commercial product Aureo) on potted plants against *P. viticola*, where it provided very low protection; no direct effect on zoospores were observed *in vitro*. Under greenhouse conditions, the activity of the PR protein glucanase was weakly increased but not enough to consider this microorganism as a resistance inducer (Harm et al., 2011). The application of an inactivated suspension of apple-epiphytic yeast *A. pullulans* on grapevine callus culture and *in vitro* plantlets increased gene expression and enzyme activity of PAL and STS, demonstrating the possibility of a resistance induction mechanism.

*Alternaria alternata*, endophytic fungus isolated from grapevine leaves, showed high efficiency against downy mildew in pre-infection applications on leaf disks of a Pinot gris cultivar. In the presence of the endophyte in infected tissues, even without direct close contact between the 2 fungi, *P. viticola* indicated structural changes, possibly indicative of toxic metabolites causing antagonistic interactions: abnormal vacuolization, accumulation of electron-dense material in the vacuoles, and appearance of necrotic haustoria. Mass spectrometry confirmed presence of low-molecular-weight toxic metabolites, belonging to the diketopiperazines family (Musetti et al., 2006).

Grapevine endophytic fungus *Epicoccum nigrum*, isolated from *P. viticola*—infected leaves, inhibited sporangial germination in greenhouse conditions by hyphal surrounding. Due to the tight contact between sporangia of *P. viticola* and hyphae of *E. nigrum*, sporangia could not break off from sporangiophores, inhibiting its spread (Kortekamp, 1997).

Several *Fusarium* species, isolated from *P. viticola*—infected plants, showed a potential to control downy mildew. *Fusarium delphinoides, F. brachygibbosum*, 2 strains of *F. pseudonygamai* and non-identified *Fusarium* sp. strain MCC 1347 were tested *in vitro*, where they coiled around sporangiophores and caused sporangial distortion of the pathogen. Enzymatic activities produced extracellular lytic enzymes, potentially involved in mycoparasytic interactions, such as glucanase, chitinase, and protease (Ghule and Sawant, 2017). This was subsequently confirmed in pre-infection study on leaf discs and in a field trial. Sporangial production on leaf disks was reduced significantly as compared to untreated control (63–76%), and incidence of downy mildew in the field was also decreased on 8–10%, while chemical control showed 19% of disease reduction. Pre- and post-infection applications of *Fusarium proliferatum* G6, an endophytic fungus isolated from grapevine leaves, reduced sporulation and further growth of *P. viticola* on leaf disks and seedlings leaves of *V. vinifera* cultivar Riesling in a growth chamber. In field trials on a susceptible cultivar, Chancellor, incidence of downy mildew after an application of *Fusarium proliferatum* G6 was reduced similarly to chemical treatment with mancozeb (Falk et al., 1996). In later studies, isolate No̱1505 of this strain was found to produce extracellular hydrolytic enzymes β-glucosidase and endo-1,4-β-glucanase that can degrade cell wall components of oomycetes (Bakshi et al., 2001).

An aqueous extract of *Penicillium chrysogenum* dry mycelium by-product from penicillin industrial production, provided high protection of grapevine plants in a pre-infection foliar application under greenhouse conditions and in field trials with natural infection. The extract reduced disease severity under different disease pressures comparable to the copper fungicide and resistance inducers BTH or BABA. Crude extracts of an endophytic fungus *Phomopsis* sp. CAFT69, isolated from the medical plant *Endodesmia calophylloides*, inhibited motility and induced subsequent lysis of *P. viticola* zoospores. Zoosporicidal compounds were isolated and characterized (Talontsi et al., 2012).

*Rhizophagus irregularis*, an arbuscular mycorrhizal fungus (AMF), altered an expression of several *P. viticola* effectors which interfered with a pathogen's ability to infect the grapevine cuttings in semi-controlled conditions (Cruz-Silva et al., 2021).

In studies against several plant pathogens, *Trichoderma harzianum* suppressed the pathogen's growth by cell wall degradation using lytic enzymes such as proteases, glucanases, and chitinases (Haran et al., 1996; Reino et al., 2008). *Trichoderma harzianum* T39 (commercial product Trichodex) reduced the development of *P. viticola* on susceptible grapevine cultivars (*Vitis vinifera* cv. Pinot Gris and cv. Pinot Noir) under greenhouse conditions by the enhanced expression of defense-related genes, priming for enhanced expression of these genes, and increased induction of protective enzymes, leading to systemic resistance to downy mildew (Perazzolli et al., 2008, 2011; Palmieri et al., 2012; Kamble et al., 2021; Lazazzara et al., 2021). Also, the treatment by *T. harzianum* HL1, isolated from the cultivated soil, increased the POX activity and reduced disease severity in naturally infected grapevine plants under field conditions (El-Sharkawy et al., 2018). Soil-born *Trichoderma viride* HL5 was effective under field conditions. After natural infection, fungal treatment increased plant peroxidase level and reduced disease severity. In addition to the biocontrol effect, quality parameters of berries (TSS/acid ratio) and plant shoot length was significantly higher than in control treatment (El-Sharkawy et al., 2018). *T. viride*, like many members of *Trichoderma* genus, is known for its high cellulase production, an important mechanism involved in pathogen suppression (Li et al., 2010).

#### Bacterial Agents

During recent decades, beneficial endophytic bacteria have emerged as promising BCAs for their ability to enter and survive inside plant tissues (Compant et al., 2005). More recently, among 239 bacterial endophytes isolated from grapevine leaves, two endophytic strains, identified as *Bacillus subtilis* GLB191 and *B. pumilus* strain GLB197 have proved their preventive effects for *P. viticola* both on leaf disks and during 2-year field trials. Treatment with these BCAs produced more efficiency than chemical control (multiple application of 1% Bordeaux mixture and fungicide Polyoxin) 25 days after the natural infection (Zhang et al., 2017). Pesticide-resistant strain *B. subtilis* KS1, that was isolated from grape skin and produced an antifungal lipopeptide iturin A, decreased the incidence of downy mildew on berries and leaves in vineyards of sensitive cultivars Koshu and Merlot, where it was applied each week during the growing season (Furuya et al., 2011). Endophytic bacterium *B. altitudinis* GLB197 isolated from grapevine leaves showed significant inhibition of downy mildew both in leaf disk test and in field experiments (Zeng et al., 2021). Isolated from *Vitis* sp. cv.Koshu, *B. velezensis* strain KOF112 inhibited zoospore release from *P. viticola* and upregulated the expression of several pathogenesis-related genes (Hamaoka et al., 2021).

Copper-resistant *Lysobacter capsici* AZ78, isolated from tobacco rhizosphere, showed efficiency against grapevine downy mildew under controlled conditions, where disease severity on leaves was reduced similarly to application of copper fungicide (Puopolo et al., 2014b; Brescia et al., 2021). Direct inhibition of the pathogen was reached by production of the secondary metabolite 2,5-diketopiperazine that was found to be toxic against sporangia of some plant pathogenic oomycetes, including *P. viticola* (Puopolo et al., 2014a; Cimmino et al., 2020). In later studies, results of bacterial genome sequencing showed presence of genes encoding production of lytic enzymes responsible for cellulose, chitin, laminarin, and proteins degradation (Puopolo et al., 2016).

Bacterial strain SY286 of *Ochrobactrum* sp., isolated from asymptomatic downy mildew—infected grapevine leaves, had a high inhibitory activity against *P. viticola* in the detached leaf assay and under field conditions. SEM observation showed that this strain is able to destroy mycelium and sporangia of the oomycete; *in vitro* analyses detected production of such metabolites as siderophores, cellulases, proteases, and amylases that are important in BCA-plant-pathogen interactions (Zang et al., 2020).

*Paenibacillus* sp. strain B2, isolated from the rhizosphere of *Sorghum bicolor* showed high antagonistic activity against *P. viticola* due to the secretion of hydrolytic enzymes or induction of plant defenses. The strain B2 inoculated with the vesicular-arbuscular mycorrhizal fungus *Glomus mosseae*, was found to produce peptide paenimyxin that significantly reduced the mobility of *P. viticola* zoospores and mycelium growth of *B. cinerea*. When an extracted peptide was applied by spraying before the infection, it was not effective, suggesting a direct action of paenimyxin (Hao et al., 2017). Endophytic bacterial strain *Pseudomonas fluorescens* PTA-CT2, isolated from grapevine stems, reduced downy mildew disease by induction of systemic resistance in pre-infection applications on greenhouse-grown plants (Lakkis et al., 2019). *P. fluorescens* Pfl, isolated from the rice rhizosphere, induced plant systemic resistance after foliar application and subsequent infection with *P. viticola*. Talc-based formulation of Pfl strain was tested under greenhouse conditions, where activity of defense enzymes such as peroxidase, polyphenol oxidase and phenylalanine ammonia lyase was found to be increased in a response to the pathogen attack (Archana et al., 2011).

Terrestrial bacterial strain *Streptomyces atratus* PY-1 significantly reduced downy mildew severity in a detached leaf assay with slightly less reduction in a field trial on a low-resistant cultivar, Centennial Seedless. SEM observations showed disruption of pathogenic sporangia and sporangiophores. Secondary metabolites inhibited *P. viticola* sexual reproduction through reduced protein synthesis (Liang et al., 2016). In a previous *in vitro* study, *Streptomyces* sp. ANK313 inhibited motility of *P. viticola* zoospores and caused its lysis by the production of secondary metabolite khatmiamycin, an ester derivative of the antibiotic juglomycin (Abdalla et al., 2011; Yoshioka et al., 2019). S. *viridosporus* HH1 reduced disease severity under field conditions with a natural infection. Levels of polyphenol oxidase, total phenol content and parameters of berry quality were significantly better than in non-treated or chemically treated plants (El-Sharkawy et al., 2018). Under the same conditions, *S. violatus* HH5 not only reduced a disease severity and increased polyphenol oxidase level, but also improved parameters of plant growth, yield, chemical composition of leaves and berries.

## Future Prospects and Conclusions

Huge progress had been made in understanding of interactions between *P. viticola* and grapevine since the recognition of this disease. Nevertheless, continued research in many aspects of these interactions should take a place to improve current control and develop new protective strategies. Modern molecular techniques offer insights into phylogenetic affiliations, pathogen susceptibility to control treatments and genetic aspects of plant resistance. Breeding new cultivars promise improved practical usage, safety, and endurance of the resistance without environmental concerns. Resulting resistant varieties that satisfy a broad range of requirements should be widely introduced in grapevine-growing regions. Furthermore, the effects of cultural practices on survival and viability of oospores requires additional study. The potential to introduce these practices offer improved effectiveness of pathogen control.

The role of collateral hosts that can act as a source of infection and the impact of weather and soil factors on asexual and sexual reproduction of pathogens should be investigated. Studying these relationships will be helpful for epidemiology and prognosis of disease development and will allow growers to choose optimal control strategies.

Integrated disease control should select the most appropriate and cost-effective techniques, while new schemes for plant protection are created. Weather and geographic conditions, resistance of cultivars, treatment with chemical and organic pesticides, use of biocontrol agents and resistance inducers all timed to pathogen stage offer the potential for significantly reduced pathogen effects and improved crop yields.

Biocontrol measures, while inherently attractive, face several challenges in practice, making this issue one of the most important directions for research moving forward. Technical problems in production, maintenance of pure culture in the case of liquid products, the search for optimal formulations suitable for farmers and their current equipment, duration and stability of products, extraction and purification of derivatives such as resistance inducers on an industrial scale are challenging and require additional research. Product registration is often complicated, expensive, and time-consuming, but necessary for safe introduction of new products to the market. Mechanisms of interactions between microorganisms and potential eukaryotic hosts should be scrupulously investigated to understand and avoid the potential production of human and mammal pathogens. Therefore, studies of interactions between BCA and the surrounding environment, for example, beneficial insects and other crops must also be considered.

## Author Contributions

KK, QE, and EA conceptualized, wrote the first draft of the manuscript, and made the figures. KK, QE, CJ, JN, CC, and EA revised and approved the final version of the manuscript. All authors contributed to the manuscript structure and discussions.

## Funding

This work was supported by the University of Reims Champagne-Ardenne, FEDER and the region Grand Est, France (VitEst project).

## Conflict of Interest

The authors declare that the research was conducted in the absence of any commercial or financial relationships that could be construed as a potential conflict of interest.

## Publisher's Note

All claims expressed in this article are solely those of the authors and do not necessarily represent those of their affiliated organizations, or those of the publisher, the editors and the reviewers. Any product that may be evaluated in this article, or claim that may be made by its manufacturer, is not guaranteed or endorsed by the publisher.
